# Resource‐Efficient Electrodes with Metallized Woven‐Glass‐Grid Current Collectors for Lithium‐Ion Batteries

**DOI:** 10.1002/cssc.202402233

**Published:** 2024-12-03

**Authors:** Yen‐Ming Li, Mohammadjafar Momeni, Huy Nguyen Dang Duc, Suvi von Bahder, Friedrich Roth, Wolfram Münchgesang, Manfred Danziger, Winfried Voitus, Dominik Nuss, Cornelia Sennewald, Tilmann Leisegang

**Affiliations:** ^1^ Institute of Experimental Physics, TU Bergakademie Freiberg Leipziger Str. 23 09599, Saxony Freiberg Germany; ^2^ elfolion GmbH Quedlinburger Str. 14 06485, Sanxony-Anhalt Quedlinburg OT Gernrode Germany; ^3^ Center for Efficient High Temperature Processes and Materials Conversion (ZeHS) TU Bergakademie Freiberg Winklerstr. 5 09599, Saxony Freiberg Germany; ^4^ Fraunhofer-Institute for Wind Energy Systems (IWES) Großer Westring 2 27592, Bremen Bremerhaven Germany; ^5^ Institute of Textile Machinery and High Performance Material Technology TU Dresden Hohe Str. 6 01069, Saxony Dresden Germany

**Keywords:** current collector, lithium-ion batteries, metallized woven-glass-grid, resource-efficiency, specific energy

## Abstract

A novel class of resource‐efficient, woven‐glass‐grid current collectors (CCs) for Li‐ion batteries is introduced. These CCs are based on ultra‐light multifilament glass threads, woven to a grid and surrounded with a thin metal layer (equivalent to a 1 μm‐thick metal foil) in a roll‐to‐roll physical vapor deposition process. This saves >90 % of the required Cu and Al metals and reduces the mass of the CCs by >80 %. At the same time, the gravimetric capacity of anodes with graphite and cathodes with LiCoO_2_ active material increases by 48 % and 14 %, respectively, while full cells are characterized by an increase of 26 %. Thus, the specific energy can be improved by 25 %. A complete anode and cathode fabrication process from preparing the CCs and electrodes to cells is described and demonstrated in coin cell format. Coin cells with woven‐glass‐grid CCs achieved 300 cycles with a capacity retention of 93 %, a Coulombic efficiency of >99.9 %, and a higher rate capability until a C‐rate of 3 C. This technology opens up new possibilities for designing ultralight CCs with dedicated surface properties for Li and beyond Li batteries.

## Graphical abstract

The use of metallized glass‐based woven‐grids instead of metal foils as current collectors for LIBs leads to a significant reduction in metal usage by over 90 % and weight savings of more than 80 %, resulting in an 11 % increase in specific energy at the cell level.

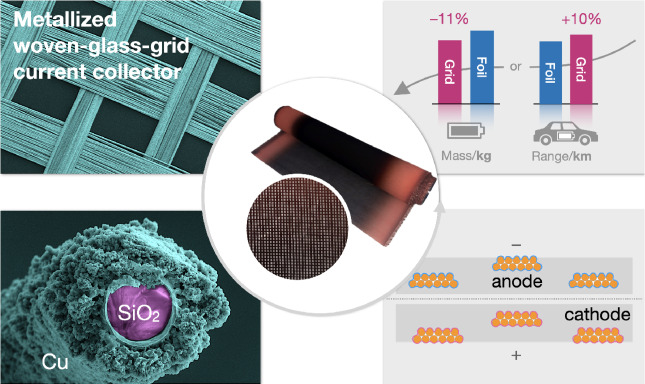

## Introduction

**Figure 1:**
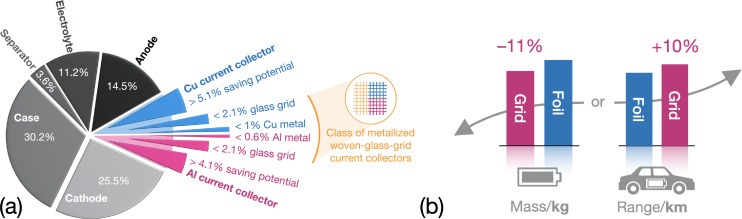
(a) Mass ratios of components in a LIB according to Ref. [1] and modified by a decomposition of the Cu (blue) and Al CC (pink) when utilizing the novel MWGG CCs. The fraction of conventional CCs (colored light‐shaded blue and pink areas) that amounts to 15 % and the potential savings that are estimated by 9.2 % when using the MWGG CCs are emphasized. (b) Benefits when using the MWGG (“grid”) CCs in an electric vehicle LIB: either 11 % mass reduction or 10 % range extension (with total battery mass remaining the same) can be expected.

Current collectors (CCs) are often considered as “dead weights” in Li‐ion batteries (LIBs). They collect and conduct liberated electrons during discharge in the anodes of LIBs and transfer them to the cathode electrodes. When charging, the reverse process occurs. Although having no electrochemical activity, CCs are indispensable parts of modern LIBs by providing mechanical support to active materials (AMs) in electrodes. In today′s conventional LIBs, Cu (density: 8.96 g cm^−3^) and Al foils (density: 2.70 g cm^−3^) are used as CC in anode and cathode electrodes, respectively, which account for (15–50) % total mass of the cell in LIBs,^[1]^ as shown in Figure 1a. To achieve LIBs with higher specific energy and energy density, it is desirable to reduce the amount of CCs used.

Since the inception of industrial‐scale LIBs production, there has been a continuous attempt in the battery community to reduce the CC thickness. These efforts led to the thickness reduction of Al (25 μm) and Cu (18 μm) foils in 1998 to 10 μm for both CCs by 2019.^[2]^ However, further reduction in CC thickness are hindered by several challenges, including rising manufacturing costs, diminished electronic conductivity, and reduced mechanical strength.^[3]^ Moreover, in 2023, the US government added Cu to its list of critical materials for the first time, aligning with similar classifications previously made by the EU, Japan, and Canada.[^4^, ^5^, ^6^] Since Cu is used in numerous energy‐related technologies, raising a serious concern about supply chain disruption in the near future. The need for higher energy‐dense batteries and the concern about the continuous supply of Cu highlight the importance of using Cu more effieciently in LIBs productions.

The goal of keeping the CCs amount in LIBs at a minimum while maintaining or improving the battery performance has been actively pursued by academic and industrial sectors.^[1]^ Many strategies like metallization of polymer, glass, and soft fabric substrates have been utilized to reduce the mass contribution of CCs in LIBs.[^7^, ^8^, ^9^] By designing novel CCs, researchers have not only perpared lighter CCs but have also enhanced their functionality, going beyond to just provide mechanical support of the AM. Ultralight, fire‐extinguishing CCs, porous CCs providing shorter path lengths for charge carriers to enhance the power performance, ultralight CCs with higher thermal conductivity, *etc*., are examples of novel CCs, which are reported in the literature.[^9^, ^10^, ^11^, ^12^, ^13^]

In this article, we describe a novel type of ultralight, resource‐efficient CC characterized by a thin layer of Cu/Al coated on woven‐glass‐grids. The glass grid substrate is cost‐effective and opens up the possibility of designing new and versatile CCs in a roll‐to‐roll (R2R) process, being favorable for industrial mass production. Moreover, since glass is classified as one of the permanent materials,^[14]^ the glass substrate has the potential of permanent recycling to further enhance the resource‐efficiency of these CCs.

Woven grids based on high‐strength filaments, such as multi‐filament glass yarns that are subsequently coated with a specialized metal envelope to meet the electronic requirements, offer an innovative and promising approach.[^9^, ^15^, ^16^] E‐glass fibers are already used for the reinforcement of materials (*e. g*. plastics) due to their beneficial price‐performance ratio.[^17^, ^18^] They provide both comparatively high tensile strength (∼3700 MPa; Cu metal: 210 MPa) and moduli of elasticity (∼77 GPa; Cu metal: 110 GPa),[^17^, ^19^] high melting temperatures (∼900 °C–1245 °C; Cu metal: 1083 °C)[^19^, ^20^, ^21^] and, to a lesser extent, elongation (∼5 %; Cu metal: 60 %).[^17^, ^19^] The finest woven‐glass‐grids, currently among the most reliably available on the market, are made from EC5 5.5 tex multifilament glass yarns, resulting in an area density of only 25 g m^−2^ (Porcher Industries Germany GmbH).^[22]^ It is worth mentioning that E is the type of glass, *i. e*. e‐glass, C is the yarn type, *i. e*. continuous, 5 is the diameter of the filaments, *i. e*. 5 μm, 5.5 is the yarn weight in tex, *i. e*. 5.5 g km^−1^. For a 9 μm‐thick Cu foil, the area density is close to 80 g m^−2^, thus more than three times larger. Technologically, it is certainly possible to weave even finer multifilament glass yarns into grids, for example, EC5 2.8 tex or EC4 1.7 tex. With these, area densities ≤10 g m^−2^ are achievable (Porcher Industries Germany GmbH).

### CCs Based on Multifilament Glass Yarns – Metallized Woven‐Glass‐Grid CCs

**Figure 2:**
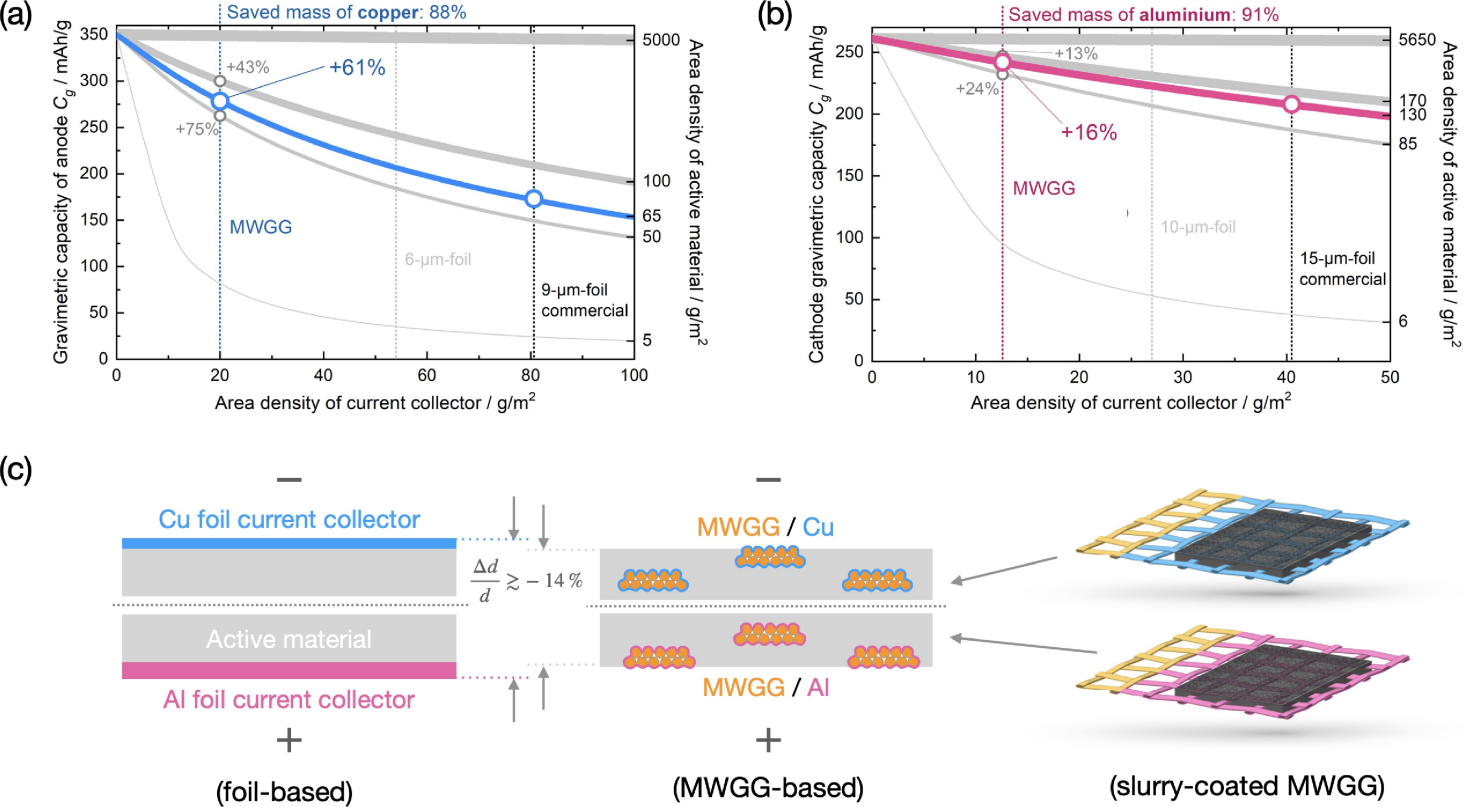
Estimation of the increase in theoretical *C*_g_ for (a) the anode normalized to graphite AM and (b) the cathode normalized to LiCoO_2_ AM. The gain in *C*_g_ depends on both the area density of the CC and the amount of AM used. The line thickness indicates the AM loading (thicker = more), with the blue (a) and pink (b) lines representing the AM loading used for commercially available electrodes (MTI Corp). (c) Schematic cross‐sectional views of electrodes with foil‐based and MWGG CCs. In between the cell stacks the estimated relative thickness reduction when using MWGG CC is indicated.

Here we introduce a novel class of metallized woven‐glass‐grid (MWGG) CCs. Although Shang *et al*.^[9]^ recently published an article on this class of CCs, the idea is significantly older^[15]^ and the first experimental work was already done before.[^16^, ^23^] The thin metal coating is significantly thinner than the thickness of commercial foil‐CCs (*e. g*. 9 μm for Cu foil‐CC)^[24]^ used in electrodes of LIBs. The MWGG CCs are not only a prerequisite for achieving higher specific energies and energy densities but they also consume significantly less valuable metals, as shown in Figure 2.

Taking into account the low area density of the woven‐glass‐grid base material and the thin metal coating, *i. e*. the CC, the gravimetric capacity *C*_g_ of an electrode (anode or cathode) is significantly affected in the first instance. We want to note two terms used later: *electrode* = CC + dried slurry, *mass of electrode* = *m*_CC_ + *m*_AM_ + *m*_binder_ + *m*_CA_. Here, *m*_CC_, *m*_AM_, *m*_binder_, and *m*_CA_ are the masses of the CC, the AM, the binder, and the conductive additive CA. The gain in *C*_g_, shown in Figure 2a, b, can be estimated starting from the theoretical *C*_g_ of the AM:

(1) $$C_{g,\;\mathrm{theo}.}=\frac{F\cdot n_{\mathrm{Li}}}{M\cdot 3600}.$$

Here, *F* is the Faraday constant, which is 96,500 C mol^−1^, *n*_Li_ is the number of Li ions per molecule of the AM (*n*_Li_ = 1 for graphite and LiCoO_2_ (LCO)), and *M* is its molar mass (*M* = 72.07 g mol^−1^ for graphite and *M* = 97.9 g mol^−1^ for LCO). Then, the *C*_g_ of the electrode (anode and cathode) can be calculated as follows:

(2) $$C_{g}=\frac{C_{g,\;\mathrm{theo}.}\cdot m_{\mathrm{AM}}}{m_{\mathrm{CC}}+m_{\mathrm{coating}}}.$$

Here, *m*_coating_ = *m*_AM_ + *m*_binder_ + *m*_CA_ is the mass of the amount of dried slurry applied to the CC. Thus, the *C*_g_ depends on four masses. We plotted the estimation of *C*_g_ as a function of *m*_CC_ for different *m*_AM_, but for the sake of generalization, we used area densities for the abscissa. For the composition of the slurry, we adapted the values from a recipe of MTI Corp.[^24^, ^25^] In Figure 2, the increasing amount of applied AM is symbolized by the increasing line thickness for the anode (Figure 2a) and the cathode (Figure 2b), respectively. The blue and pink curves represent the amount of AM used in commercially available electrodes from MTI Corp. The area density of the commercially available Cu and Al foils (used as CCs) as well as of the MWGG CCs used here are indicated (black, blue, and pink vertical dashed lines). As can be seen, the used MWGG CCs provide a theoretical increase in *C*_g_ by a factor of 1.61 and 1.16 for the anode and cathode, respectively, compared to the commercial electrodes from MTI Corp. Simultaneously, the mass of Cu and Al metal is reduced by 88 % and 91 %, respectively. As a result, a *full cell* composed of an anode and a cathode with an MWGG CC would be characterized by an increase in gravimetric capacity normalized to both electrodes *C*_g,both_ by a factor of 1.33 due to the saved mass of 73 % of the CCs (see Figure S1).

In terms of the energy density, the use of MWGG CCs allows a reduction in electrode thickness, with the maximum reduction being determined by the absence of CCs, which is 23 %, and thus increasing the energy density of a LIB accordingly by 30 %. The ideal values we can obtain here are a 14 % thickness reduction and a 17 % increase in energy density. We would like to note that the estimation of the energy density at cell level (only the electrodes were considered) was calculated based on the area capacities of commercial electrodes (MTI Corp).

A high mechanical strength of MWGG CCs is expected due to the use of glass. The special surface morphology of the metal coating is considered to improve the slurry adhesion strength. Moreover, the grid penetrates and pervades the electrode and so optimizes the travel path lengths of the charge carriers (electrons/ions).^[10]^ We would like to emphasize at this point that the MWGG CCs are characterized by the combination of conductive linear elements that form a grid in contrast to an areal conducting foil. The subsequent electrode is therefore not characterized by an “AM on a foil”, but by an “embedded” CC that penetrates the AM, providing enhanced funtionality. . Extra benefits of using MWGG CCs that are not explored in this study are: (1) the electrolyte should better penetrate the electrode and decrease wetting times, (2) high thermal stability due to the use of glass that is non‐combustible.^[9]^

In summary, the application of MWGG CCs predominantly leads to an increase in *C*_g_, while the mass of Cu and Al is significantly reduced. To give an impression of the numbers let us consider a 100 kW h battery pack used in a Tesla Model 3. It has an estimated mass of 355 kg at cell level.^[26]^ Thus, this battery would either be 39 kg (11 %) lighter or provide a range extension of 69 km (10 %) if the mass is maintained, as shown in Figure 1b. This would correspond to an increase in specific energy from about 280 W h kg^−1^ to 316 W h kg^−1^. The CC mass saved can be estimated by the values given in Figure 1a and Figure 2a, b. The battery driving range was calculated using data from Ref. [27]. Further details of the calculation are described in the Supporting Information, Note S1.

## Results and Discussion

### Microstructure, Morphology, and Mechanical Stability of the CC

**Figure 3:**
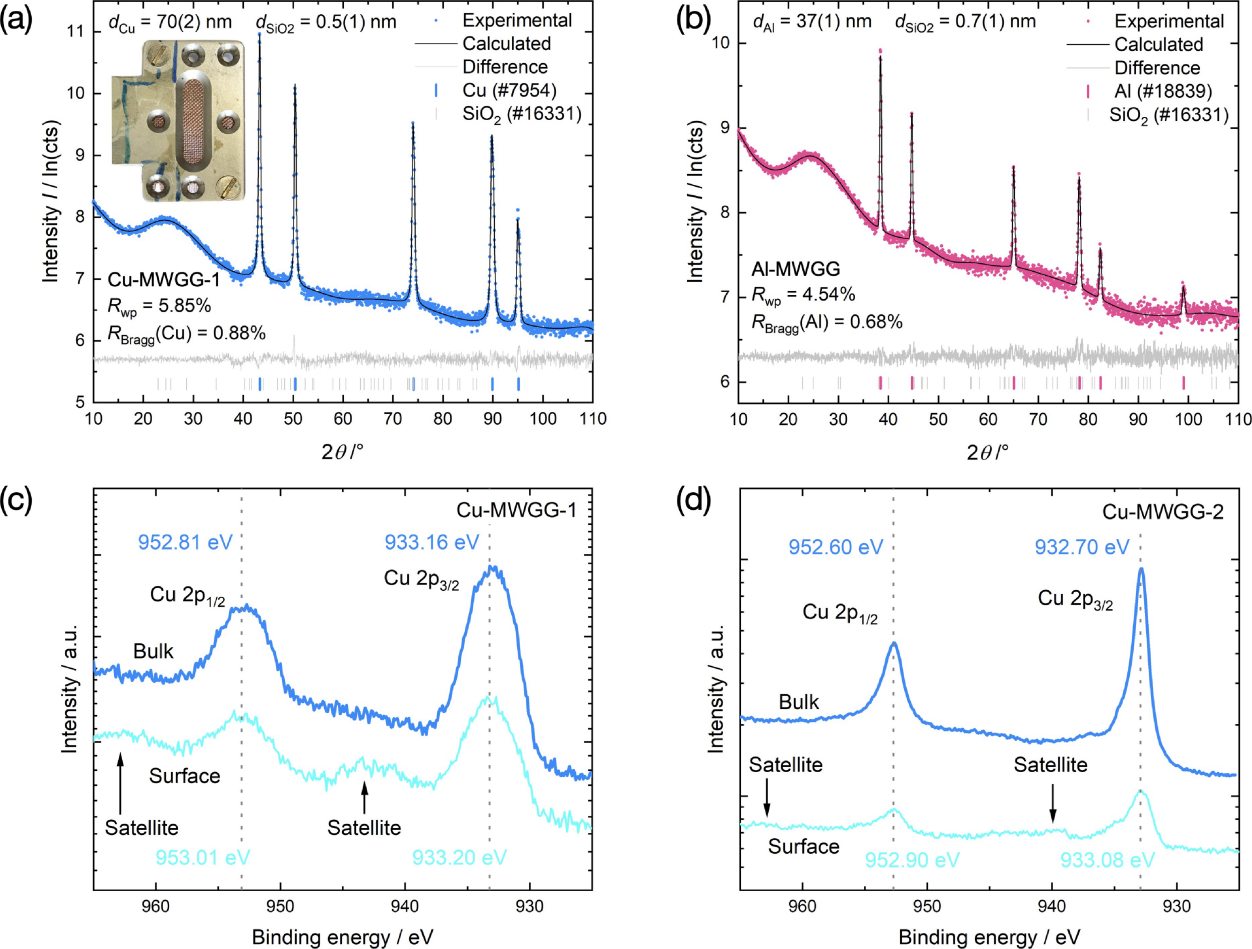
X‐ray diffraction patterns (log. scaling) of the Cu‐MWGG‐1 CC (a) and Al‐MWGG CC (b). Shown are experimental XRD data (dots), fitted curves (black line), and difference curves (gray line) along with the reflection positions of the Cu, Al, and SiO_2_ phases (the collection code from the ICSD^[28]^ is given in brackets). The modeled crystallite sizes of the Cu, Al, and glass (SiO_2_) phases, *d*_Cu_, *d*_Al_, and $d_{{\mathrm{SiO}}_{2}}$ , and refinement quality indicators *R*_wp_ and *R*_Bragg_ are given. The transmission sample holder with Cu‐MWGG‐1 CC in the middle is shown as an inset in (a). In (c, d) the XPS data before and after sputtering the surface of Cu‐MWGG‐1 (c) and Cu‐MWGG‐2 CC (d) are shown. The Cu 2p_1/2_ and Cu 2p_3/2_ are indicated.

In Figure 3a, b, X‐ray diffraction (XRD) patterns of the as‐prepared Cu‐MWGG and Al‐MWGG CC are shown. It can be seen that pronounced reflections only occur for the Cu and Al coating. Reflections from crystalline oxides were not observed (note the logarithmic scaling) or were very weak (Cu‐MWGG‐2 CC, Figure S2; an amount of 0.4 wt % oxide on the Cu coating is estimated). The C coating is not visible for the Cu‐MWGG‐2 CC due to its small amount and low scattering ability. Hence, either crystalline reflections or amorphous maxima of the C coating would be covered by the background signal (see Supporting Information for further details, Note S2). The weak and broad maximum at 2*θ* = 26° results from amorphous SiO_2_ (the underlying woven‐glass‐grid). Note that due to the low atomic number of Al, the reflection intensities for Al are lower than those for Cu, so that the maximum due to SiO_2_ appears more pronounced.

The phase purity was quantitatively characterized by Rietveld refinement. Phase‐pure Cu and Al phases were determined for the Cu‐ and Al‐MWGG CCs, also indicated by the low refinement quality parameters *R*_wp_ and *R*_Bragg_. The determined crystallite sizes *d*_Cu_, *d*_Al_, and $d_{{\mathrm{SiO}}_{2}}$ for the Cu and Al coatings, and the SiO_2_ from the glass filaments were 62(1) nm, 57(5) nm, and 0.5(1) nm, respectively.

From X‐ray photoelectron spectroscopy (XPS) analysis, the surface as well as bulk phase composition of the Cu foil (Figure S3a) and the Cu‐MWGG CCs (Figure 3c, d) was determined by *in situ* sputtering. Besides Cu and C (protection layer), O and N were found, which are attributed to Cu oxides and the nitrogen atmosphere during Cu‐coating of the woven‐glass‐grids, respectively.

For the reference Cu foil‐CC (Figure S3a), we observed significantly shifted peaks at the surface (compared to pure Cu metal) at 934.60 eV and 954.58 eV, which are connected to Cu 2p_3/2_ and Cu 2p_1/2_ states, respectively.^[29]^ The collection of strong satellite peaks between (940–945) eV and (960–965) eV shows that at the surface only CuO exists (Cu(II) oxide).[^30^, ^31^] This is congruent with Ref. [32], which states that first CuO is formed at the surface of Cu during the oxidation process. With sputtering, the observable Cu 2p_3/2_ and Cu 2p_1/2_ peaks suggest the presence of pure Cu in the bulk with a little amount of Cu_2_O (see weak satellites in logarithmic scaling).

For the Cu‐MWGG‐1 CC (Figure 3c), the broad Cu 2p_3/2_ and Cu 2p_1/2_ peaks at 933.20 eV and 953.01 eV, respectively, also suggest the presence of Cu(II) oxide phase at the surface. The broad and relatively intense shake‐up satellite peak between (940–945) eV and (960–965) eV and a small peak shift (in comparison to the bulk signal and consistent with the Cu foil data) support the presence of Cu(II) phase. With sputtering, the shake‐up peak vanishes and the Cu 2p_3/2_ and Cu 2p_1/2_ remain as the only peaks, which indicates that in the bulk pure Cu exists. The strong broadening of the peaks is attributed to extrinsic broadening from sample charging effects^[33]^ due to higher resistance of the Cu‐MWGG‐1 CC. Indeed, the macroscopic sheet resistance measured in 4‐point geometry is 8.00 Ω, which is nearly two orders of magnitude higher compared to Cu‐MWGG‐2 CC with 0.03 Ω.

However, we attribute the broadening to be particularly caused by the higher fraction of illuminated glass substrate with a resistivity of >10^12^ Ω cm. The higher fraction of illuminated glass substrate is due to the specimen preparation process of Cu‐MWGG‐1 CC: the lower amount of Cu and the small specimen area of only several mm^2^ lead to an overall reduced stability of the MWGG, and the filaments themselves separate, so that more uncoated glass appears.

**Figure 4:**
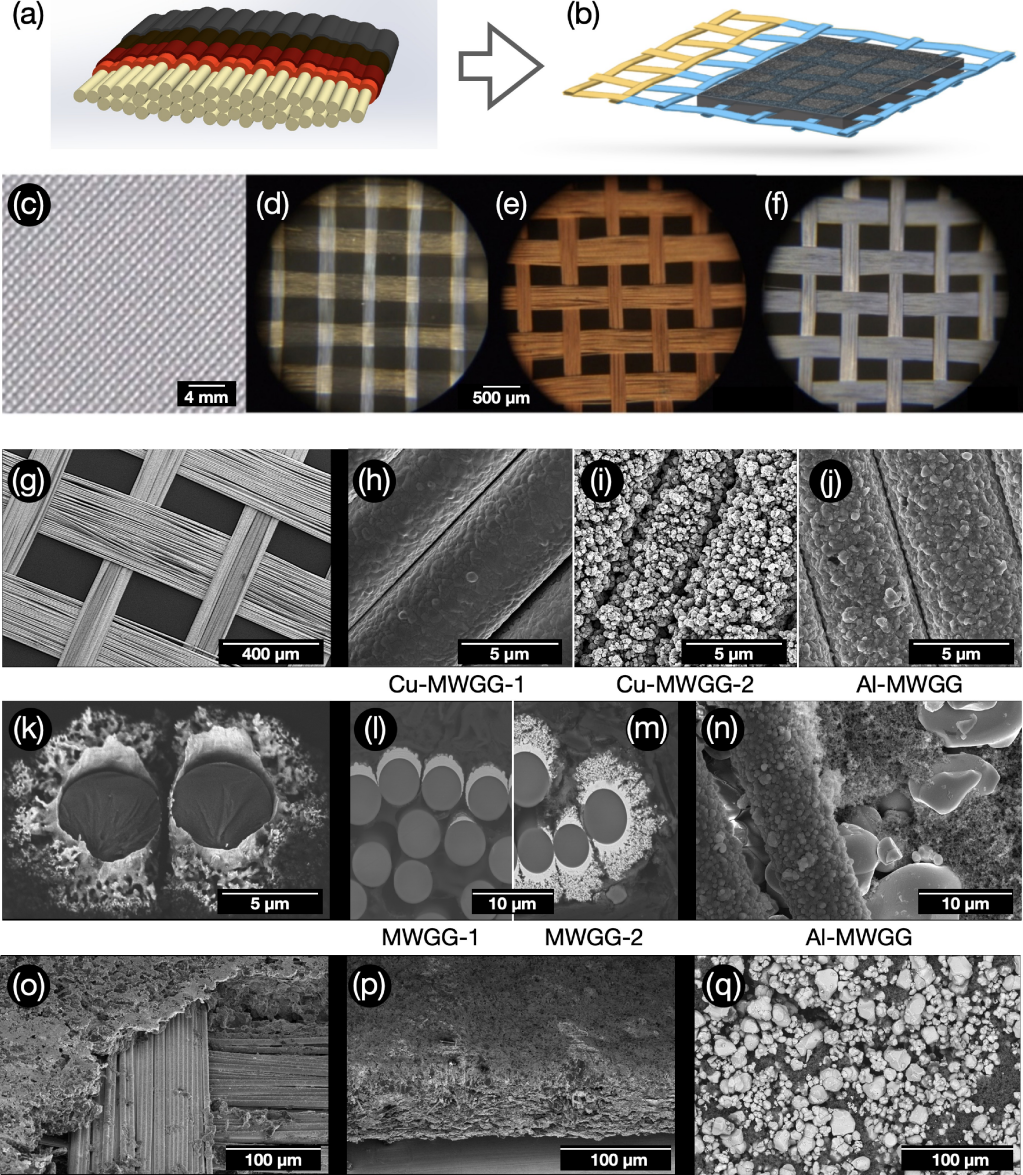
Schematic representations of the metallized glass filaments (yarn) (a) and the slurry‐coated anode MWGG CC (b). Shown are the morphology and microstructure at different levels of the manufacturing process: (c, d) woven‐glass‐grid base material, (e–k) MWGG CCs, (l–q) electrodes. In (g–j, n–p) electron micrographs with secondary electrons are shown, while in (k–m, q) all electrons were detected (material contrast). The different micrographs show: (e, g, h) Cu‐MWGG‐1 CC, (i) Cu‐MWGG‐2 CC, and (f, j) Al‐MWGG CC, (k) two Cu‐coated glass filaments in a cross‐section of Cu‐MWGG‐2 CC (light structures are Cu, round dark gray objects are glass filaments), (l, m) cross‐sections of the electrode with: (l) Cu‐MWGG‐1 and (m) Cu‐MWGG‐2 CCs, (n) the back side of the LCO electrode with Al‐MWGG CC, (o) anode based on Cu‐MWGG‐1 CC before and (p) after calendering, and (q) LCO cathode (top view; light particles are LCO).

For the Cu‐MWGG‐2 CC (Figure 3d), at the surface the Cu 2p_3/2_ and Cu 2p_1/2_ peaks were observed at 933.08 eV and 952.90 eV, respectively. Here, only a low‐intense shake‐up satellite peak exists between (940–945) eV as well as a broadening and a small peak shift of both Cu peaks compared to the bulk data. This indicates a small amount of CuO on the surface, consistent with foil data and Ref. [32]. Although Cu‐MWGG‐2 CC has a higher surface area due to the fractal structure (Figure 4i, k, m) the amount of CuO is qualitatively not higher in comparison to the Cu‐MWGG‐1 CC and especially to the Cu foil. This is attributed to the protecting C layer. However, for the Cu‐MWGG‐2 CC we observed a minor amount of Cu_2_O by XRD. We attribute this to the specific morphology: for XPS the signal mostly stems from the fractal Cu surface, while for XRD it mostly stems from the bulk near the glass filaments’ surface. We note that the full‐width at half‐maximum (FWHM) of the bulk XPS Cu peaks are narrower than the surface peaks which is consistent with a less oxidized Cu in the bulk.

For the Al‐MWGG CC, we also demonstrated the similarity with an Al foil (see Figure S3b for details).

The morphology of the MWGG CCs and the metal coating can be seen in Figure 4, which shows optical micrographs and scanning electron microscopy (SEM) images with different magnifications. The interwoven glass yarns consisting of single filaments and the coated ones by Cu or Al can be seen in Figure 4c–k. At higher magnification, a fractal‐like metal surface is visible for the Cu‐MWGG‐2 CC (Figure 4i), which can be seen more clearly in the cross‐section of Cu‐MWGG‐2 CC (Figure 4k). With *fractal*, we want to emphasize that our vacuum‐engineered highly porous metal solid‐state structures with a large surface area are produced by a dedicated additive manufacturing vacuum process that is characteristic for the generation of fractal structures (see Supporting Information for more details, Note S3). In comparison, Cu‐MWGG‐1 CC (Figure 4h, l) has a smooth surface morphology without fractal structures. For the Al‐MWGG CC (Figure 4j), the metal surface is characterized by a roughness that is higher than for Cu‐MWGG‐1 CC, although its morphology is not fractal. The fractal character and the specific morphology of the metal coating contribute to a high surface area, as evidenced by Brunauer–Emmett–Teller (BET) measurements. The determined surface area is 0.74 m^2^ g^−1^ and 1.24 m^2^ g^−1^ for Cu‐MWGG‐1 and Cu‐MWGG‐2 CC, respectively, which is about two orders of magnitude higher than the geometrical surface area of a Cu foil (0.01 m^2^ g^−1^). We assume that the value for the Al‐MWGG CC lies in between the values of the Cu‐MWGG CCs. According to Ref. [34], a larger surface area of the CC improves the contact between the dried slurry mass and the CC and reduces the interface resistance. We refer to both below when describing adhesion strength test and impedance spectroscopy results. As indicated by Figure 4m, the dried slurry is intervened with the Cu structures. Figure 4n–q show the SEM images of the MWGG‐based electrodes.

**Figure 5:**
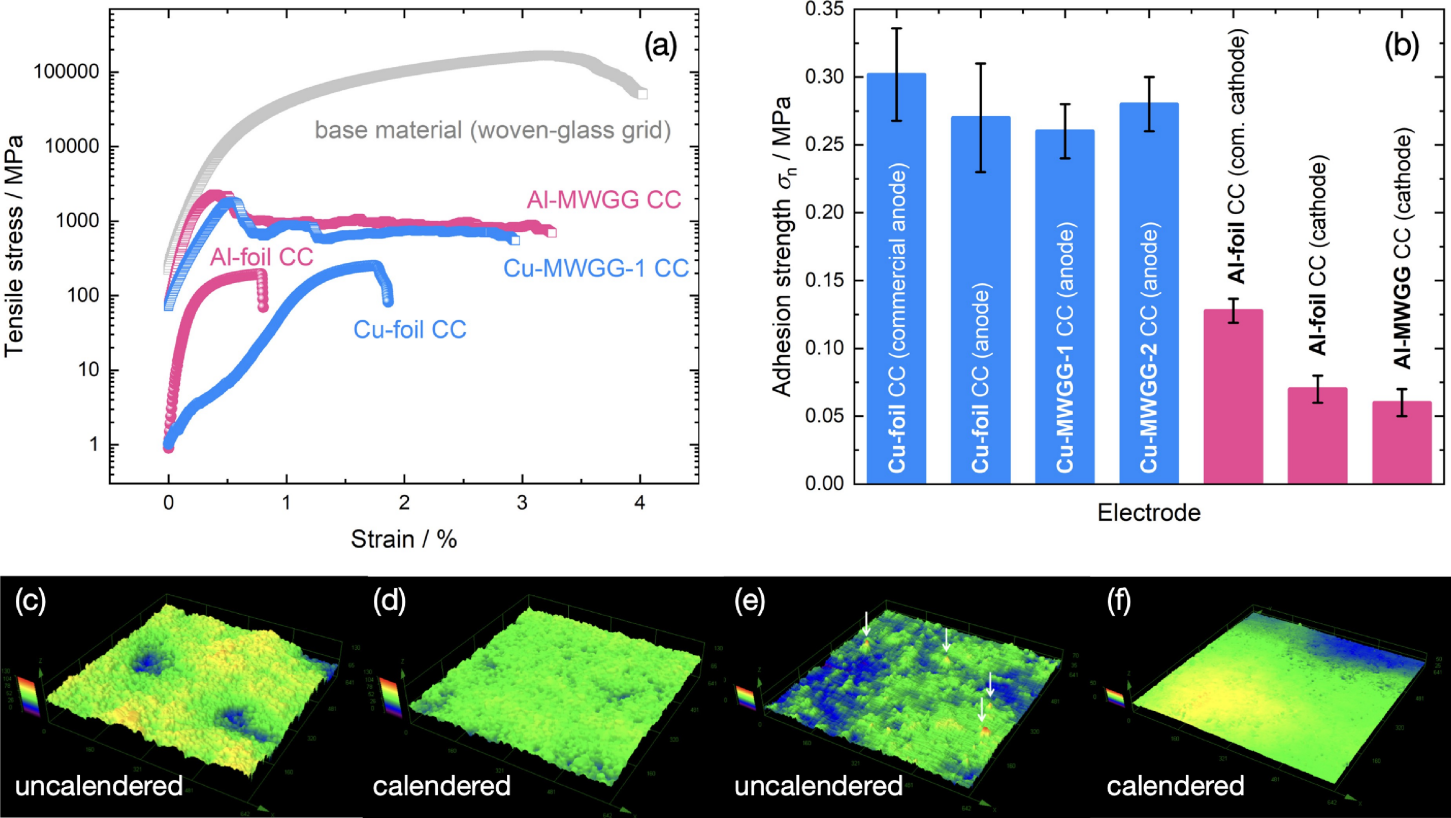
(a) Tensile stress in dependence on strain obtained from stripe tensile tests. Note the logarithmic scaling of the ordinate. (b) Adhesion strengths *σ*_n_ of the dried slurry mass on the Cu CCs (blue) and Al CCs (pink). (c–f) Height profiles of the uncalandered and calandered anode with Cu‐MWGG‐1 CC (c, d) and cathode with Al‐MWGG CC (e, f), taken with a confocal microscope. The false color scale covers a value range from 0 μm to 130 μm (c, d) and 0 μm to 50 μm (e, f). An area of 643 μm × 642 μm is shown. The white arrows in (e) indicate reflections from LCO particles.

Tensile tests for determining the mechanical stability were carried out on the different CCs and the MWGG CC base material (woven‐glass‐grid). The results are shown in Figure 5a. Here, the tensile stress was calculated according to the *real* area of the cross‐section of the samples/CCs, *i. e*. the geometrical cross‐sectional area for the metal foils (Cu foil: 0.5 mm^2^, Al foil: 0.7 mm^2^) and the cross‐sectional area resulting from the individual 111 filaments of the yarns (Figure 4a) forming the cross‐section (Cu‐/Al‐MWGG CCs: 0.007 mm^2^, base material: 0.002 mm^2^). When our data are compared to the data of Ref. [9], the MWGG CCs here are characterized by one order of magnitude higher maximum tensile stress (Cu‐MWGG‐1: 1823(429) MPa, Cu foil: 252(50) MPa, Al‐MWGG: 2313(571) MPa, Al foil: 195(43) MPa) and thus higher mechanical stability. This holds also for the comparison of MWGGs and foils. The high value of tensile stress of the base material (182(9) GPa) is due to the applied sizing, which acts as a mediator between the filaments via frictional forces. Therefore, the stability is higher than the MWGG CCs where the sizing was removed during physical vapor deposition (PVD) processing. The maximum strain for Cu and Al CCs (Cu‐MWGG‐1: 4(3) %, Cu foil: 2,0(1) %, Al‐MWGG: 2(2) %, Al foil: 0.6(3) %) is also higher for the MWGG CCs by a factor of >1.5 (the value for the base material is 5.2(1) %). It should be noted that for textiles (woven fabrics, meshes, grids) the conventional representation is not the tensile stress but the standard force in dependence on strain (Figure S4), which better characterizes the mechanical stability with regard to the later processing of the fine MWGG CCs in a R2R process. The standard force of the MWGG CCs (∼3 N cm^−1^) is still one order of magnitude lower than that of the foils (∼30 N cm^−1^). This is being addressed in a second generation of MWGG CCs in which the nodes of the grid will be specially fixed during PVD processing.

In summary, we realized three different MWGG CCs: Cu‐MWGG‐1 and Al‐MWGG CCs with a basic metal coating, and the Cu‐MWGG‐2 CC with a fractal morphology of 500 nm self‐affine metal structures with minor amounts of CuO (0.4 wt %) on the surface and Cu_2_O in the bulk near the glass filaments. They are characterized by a high surface area of up to ∼1 m^2^ g^−1^, high tensile strength of ∼2000 MPa, maximum strain of ∼3 %, maximum standard force of ∼3 N cm^−1^, and minimum sheet resistance of 0.03 Ω^−1^.

### Microstructure, Morphology of the Electrodes, and Adhesion Strength of the Dried Slurry

The MWGG‐based electrodes were examined by confocal microscopy before and after calendering (Figure 5c–f). The calendering reduced the surface waviness by >80 % to <6.5 μm and the surface roughness by >55 % to <4.6 μm for the anodes. For the cathodes, however, due to disturbing reflections of the LCO particles only the surface roughness could be characterized. Hence, the calendering reduced the surface roughness by >66 % to <1.3 μm. The surface roughness is mainly generated by the AM particles (Figure 4o, q).

Next, the mechanical adhesion strength *σ*_n_ of the dried slurry mass on the CCs was determined for the MWGG‐based electrodes, the foil‐based reference electrodes (processed similarly to the former), and the commercial electrodes. Example raw curves are presented in Figure S5. The results are shown in Figure 5b. It can be seen that the anodes (blue) have a comparable adhesion strength within one *σ*, where *σ* is the standard deviation of tested four samples. It should be noted that for the calculation of adhesion strength, we used the geometrical area.

The commercial anode with a loading of 2.2 mA h cm^−2^ showed the highest adhesion strength (0.30 MPa) among the anodes. The self‐coated anodes with a loading of ∼3 mA h cm^−2^ exhibited comparable adhesion strength (Cu foil: 0.27 MPa, Cu‐MWGG‐1: 0.26 MPa, and Cu‐MWGG‐2: 0.28 MPa). However, the electrode with the Cu‐MWGG‐2 CC had a slightly higher adhesion strength than the electrode with the Cu‐MWGG‐1 CC. Here, the higher surface area due to the fractal‐type morphology of the metal coating and the C coating of Cu‐MWGG‐2 CC is suggested to be the reason.

Regarding the cathodes (Figure 5b, pink), the highest adhesion strength (0.13 MPa) was also obtained for the commercial cathode with a loading of 1.9 mA h cm^−2^. The self‐coated foil‐ and MWGG‐based cathodes with a loading of ∼3 mA h cm^−2^ showed comparable adhesion strengths within one *σ*: 0.08 MPa and 0.06 MPa, respectively. The lowest adhesion strength, however, was found for the MWGG‐based cathode. We also attribute this to a lack of fractal morphology of the Al layer and a missing C layer.

The difference from the commercial electrodes is suggested to be due to the different loading, slurry composition, and (unknown) drying and calendering processes. This was already reported by, *e. g*., Haselrieder *et al*.,^[35]^ who showed that the adhesion strength varies with the composition of the slurry and decreases linearly with increasing mass loading which is also observed here. In addition, with MWGG‐based electrodes especially, we have to take into account not only the adhesion (“slurry on metal coating”) but also the cohesion (“slurry on slurry”) components that arise due to the meshes (holes) filled with dried slurry mass (Figure 2c).

In summary, the electrodes based on MWGG CCs exhibited an equivalent adhesion strength (anode: 0.28 MPa, cathode: 0.06 MPa) compared to the ones based on foil‐CCs (anode: 0.27 MPa, cathode: 0.08 MPa) and a waviness of <6.5 μm. We assume that the larger contact area due to the fractal‐type morphology of the metal coating and the C coating of Cu‐MWGG‐2 CC improve slurry adhesion.

### Electrochemical Stability of the CCs

**Figure 6:**
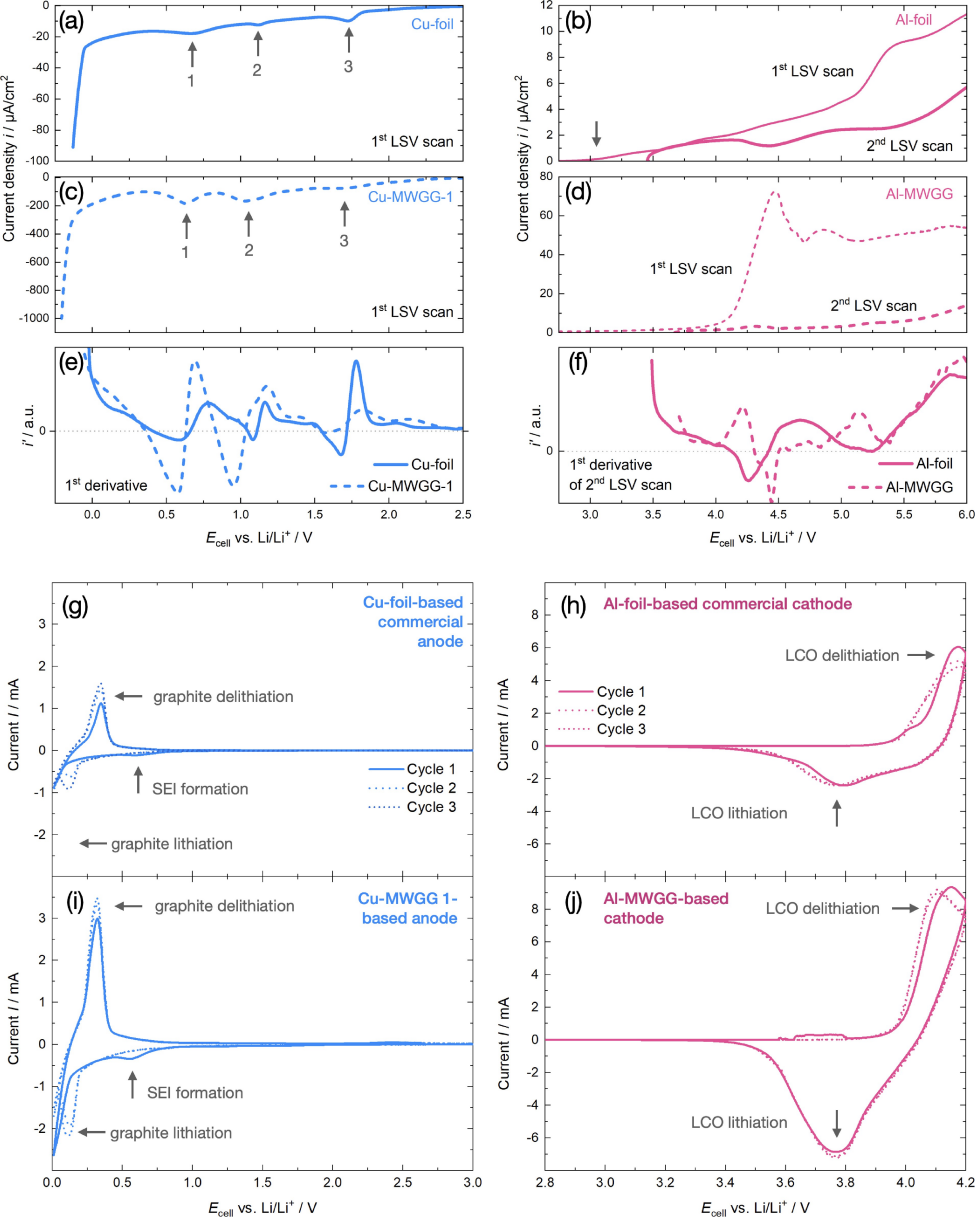
Corrosion behavior of the different CCs in 1 M LiPF_6_ salt mixed with DEC and EC (1 : 1) investigated by LSV: (a) Cu foil, (b) Al foil, (c) Cu‐MWGG‐2 CC, (d) Al‐MWGG CC, (e) first derivative of the current densities of (a, c), and (f) first derivative of the current densities of the second LSV sweep of (b, d). We note that for calculating current densities, we used the geometrical electrode area. CV curves for the first three consecutive cycles of different cells using Li metal as one electrode and a commercial anode (g) and cathode (h) as well as the MWGG‐based anode (i) and cathode (j). Characteristic extremals are indicated by gray arrows (see text for details).

The linear sweep voltammetry (LSV) test with Li metal as reference electrode was performed to investigate the electrochemical stability of the MWGG CCs in the electrolyte environment of a LIB and to compare it with the behavior of commercial Cu and Al foil‐CCs (Figure 6a–f). Of particular interest was the stability of the metal coating on the glass filaments and the influence of the glass itself.

In the case of the Cu foil (Figure 6a), in total three local minima can be seen. The minima “2” and “3” in the potential range of (1–2) V are attributed to the reduction reactions of the Cu oxide species from the surface of the Cu foil with Li and the reduction process of electrolyte components leading to the formation of the solid electrolyte interphase (SEI) layer.[^36^, ^37^] The minimum “1” between (0.50–0.75) V is due to underpotential deposition (UPD) of Li on the Cu surface.^[38]^ The sharp current increase at negative voltages arises from Li plating on Cu.^[39]^

For the Cu‐MWGG CC (Figure 6c), the general shape and position of the local minima of the LSV scan are similar to those of the Cu foil (Figure 6a), but the current densities of the electrochemical reactions are one order of magnitude larger. This is due to the large surface area of the MWGG CCs (see “Microstructure, morphology, and mechanical stability of the CCs”); from BET measurements follow that MWGG CCs have a surface area, which is two orders of magnitude higher than their geometrical area. However, we need to consider more influences here: (*i*) the Cu foil itself has a surface morphology that results in an actual larger surface area than the geometrical surface, which contributes to the increase in current density, and (*ii*) the BET values were determined by using gas absorption, which is different to the ability of the liquid electrolyte with its surface tension to penetrate all the pores, thus reducing current density.

In the case of the Al foil (Figure 6b), it can be seen that the corrosion current starts to increase after 3.1 V and continues to increase until the end of the scan. This current response with increasing voltage is due to the formation of a passivation film on the Al foil under the electrochemical conditions of the cell (AlF_3_ passivation film formation in the presence of LiPF_6_ salt).[^38^, ^40^] For the second LSV scan, the current decreases by about 50 %, indicating that the passivation process is almost completed after the first voltage sweep.

A similar behavior is observed for the Al‐MWGG CC (Figure 6d), but with a larger current response. The larger current density is again attributed to the actual larger surface area of the Al coating of the Al‐MWGG CC (see “Microstructure, morphology, and mechanical stability of the CCs”). The current density of the second LSV scan is significantly lower, which in turn is due to the completion of Al passivation.

Figure 6e, f show the first derivative of the LSV scans of Cu and Al CC. The very similar characteristics (*e. g*. zero points of the curves) prove that the MWGG CCs behave similarly to foil‐CCs under the electrochemical conditions of a LIB and in accordance with literature. Additional corrosion reactions that point to the influence of the glass substrate were not found.

#### Cyclic Voltammetry

The lithiation and delithiation process of the prepared MWGG‐ and foil‐based electrodes were characterized by cyclic voltammetry (CV) with Li metal as the reference electrode (Figure 6g–j). The same behavior was observed for both the commercial electrode with a Cu foil‐CC (Figure 6g) and the electrode with the Cu‐MWGG‐1 CC (Figure 6i). In the case of the Cu CCs with graphite AM, the first anodic peak at about 0.32 V is attributed to graphite delithiation, while the first cathodic minimum between 0 V and 0.2 V refers to lithiation. The reductive minimum at about 0.6 V is associated with SEI formation. The CV curves and characteristics are comparable to those of Ref. [41] using the same electrolyte and scan rate. The measured current value of the commercial anode is higher compared to the literature, which we attribute to the different graphite electrode properties (*e. g*. electrode size, composition, porosity, AM particle size).

CV curves for the Al CCs with LCO AM, *i. e*. the commercial electrode with an Al foil‐CC (Figure 6h) and the electrode with the Al‐MWGG CC (Figure 6j), are also similar. Two main characteristic peaks were observed for LCO delithiation at about 4.17 V (anodic peak) and lithiation at about 3.77 V (cathodic peak), which is comparable to the results reported in Ref. [42]. Although the authors in Ref. [42] used a sampling voltage of up to 4.5 V and a three‐electrode configuration, the main features of the curve are similar to our results.

We would like to point out that all peaks of the MWGG‐based electrodes are shifted to lower voltages by >10 mV. We attribute this to the larger surface area characteristic of the MWGG CCs, which leads to lower current densities and thus lower overpotentials. We also attribute the higher currents of the cells with MWGG‐based electrodes (Figure 6i, j) to the higher surface area of the MWGG CCs.

In summary, the CV shows that the MWGG‐based electrodes perform at least similarly to commercial foil‐based ones under the LIB working conditions.

#### Rate Capability Tests

**Figure 7:**
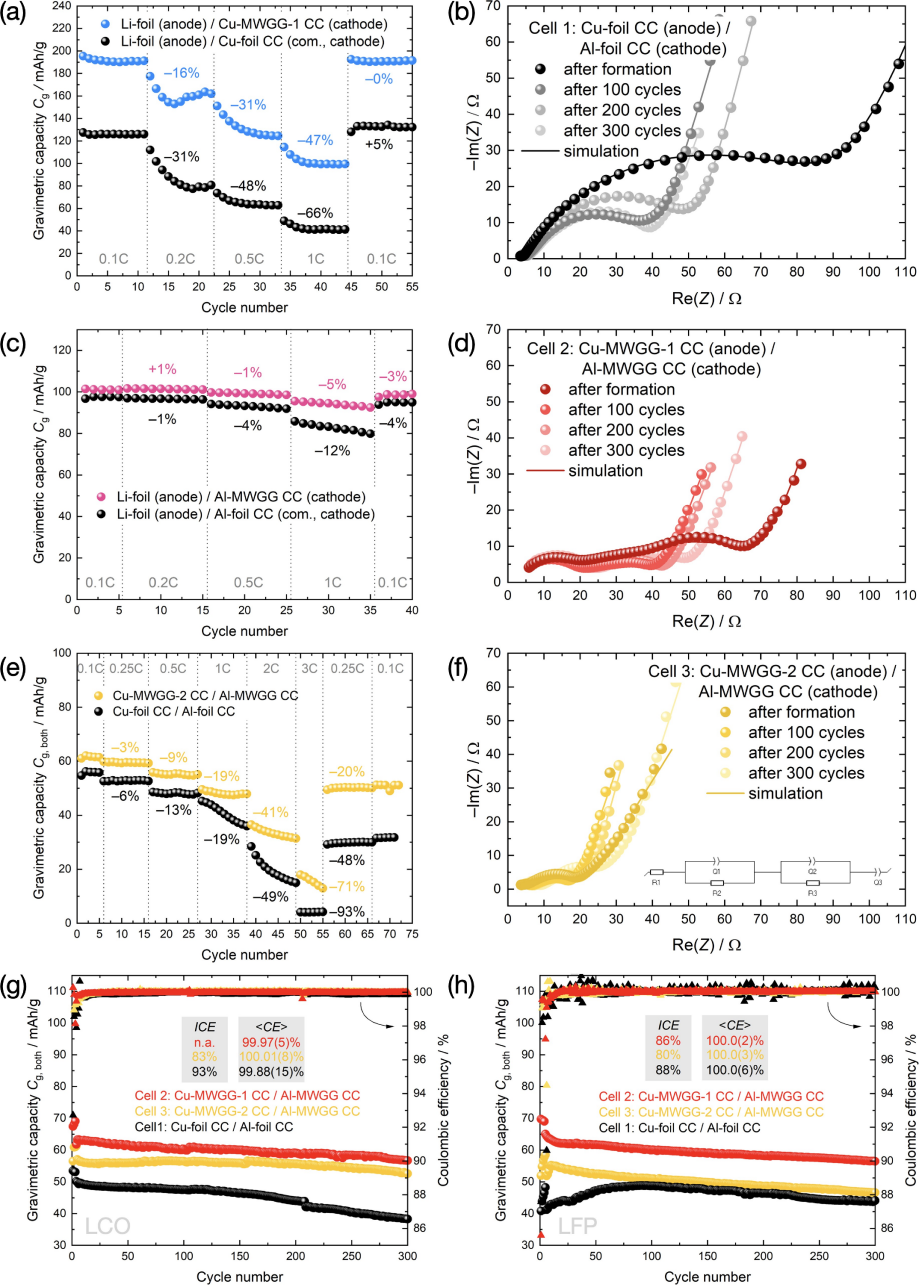
Results of the electrochemical tests: (a, c, e) the C‐rate tests of (a) graphite/Li, (c) LCO/Li, and (e) graphite/LCO cells are shown. (b, d, f) show the Nyquist plots after formation, 100, 200, and 300 cycles for three different LCO‐based cell‐types shown in (g): cell 1 (black) with foil‐based CC (b), cell 2 (red) with Cu‐MWGG‐1 CC (d), and cell 3 (yellow) with Cu‐MWGG‐2 CC (f). (g, h) present the discharge *C*_g,both_ and Coulombic efficiency data (*ICE* and <*CE*> are the initial and averaged values) for LCO‐ and LFP‐based cells. The equivalent circuit used for modeling the impedance data is shown as an inset in (f). The abbreviation “com.” stands for commercial.

Figure 7a, c show the rate capability tests of different MWGG‐based and commercial reference electrodes assembled with Li metal as the reference electrode. As the working electrode, LCO AM was used. The *C*_g_ was normalized to the electrode mass. Different C‐rates were recorded. For the commercial electrodes with foil‐CCs (black) and with MWGG CCs, Cu‐MWGG‐1 (blue) and Al‐MWGG (pink), as expected, there is an increase in *C*_g_ for the cells with MWGG‐based electrodes and a general decrease in the achievable capacity with increasing C‐rate for all electrodes. However, the cells with Al CCs showed a more stable cycling behavior, which can be attributed to the SEI layer on the graphite electrode surface.[^43^, ^44^] The rate capability of the MWGG‐based electrodes is higher than those of the commercial reference. The *C*_g_ at 1 C reached 53 % and 95 % of the *C*_g_ at 0.1 C for the electrodes with Cu‐MWGG‐1 and Al‐MWGG CC, respectively, while the commercial electrodes with Cu and Al foil‐CC only exhibited 34 % and 88 %, respectively.

The data of the *full cell* with LCO AM (Figure 7e) also indicate a higher gravimetric capacity *C*_g,both_ (normalized to both electrode masses) and a higher resilience to higher C‐rates for the cells with MWGG CCs. The cell with Cu‐MWGG‐2/Al‐MWGG‐based electrodes (yellow) showed a more stable cycling behavior from 1 C onwards. The cell with self‐coated foil‐based electrodes (black) achieved 7 % of the initial *C*_g,both_ value at 0.1 C after a C‐rate of 3 C. In comparison, the cell with MWGG‐based electrodes showed 29 % of the initial *C*_g,both_ at the same C‐rate.

The enhanced rate performance of the MWGG‐based electrodes is suggested to be due to the following: (*i*) higher interface area between AM and CC and thus lower current densities, (*ii*) the embedding of the MWGG CC into the slurry and thus shortening of charge carrier path lengths (anode: −7 %, cathode: −15 %; see Supporting Information for details, Figure S6), and (*iii*) C protection layer for the Cu‐MWGG‐2 CC. According to Refs. [45, 46], the C coating on the CC should contribute to improving the rate capability.

An imperative factor to affect the rate capability of the electrodes is the resistance of the CCs. For the Cu CCs, the sheet resistances are 8.000 Ω, 0.030 Ω, and 0.045 Ω for the Cu‐MWGG‐1, Cu‐MWGG‐2, and Cu foil‐CCs, respectively. The Al CC showed an increase of sheet resistance by 8 % (0.054 Ω) for the Al‐MWGG CC in comparison to that of the Al foil‐CC (0.050 Ω). Therefore, from the 4‐point probe measurement, we can conclude that the sheet resistance of the CCs is not the major factor to influence the rate capability of the cells here. In addition, the electrode porosity φ should be another important factor for the rate capability.

To estimate the φ of the electrodes, the volume of electrodes without pores needs to be calculated. The mass of each component in an electrode can be calculated by the multiplication of the measured electrode mass with the respective mass ratio. Then, the volume of each component can be determined by dividing the masses by their densities. Finally, the volume contribution of the CC can be subtracted, especially for the MWGG CCs. Once the total volume of the solid content in the dried slurry is obtained, the φ of the electrode can be calculated:

(3) $$\varphi =\frac{V_{\mathrm{slurry}}-V_{\mathrm{solid}}}{V_{\mathrm{slurry}}}.$$

*V*_slurry_ and *V*_solid_ are the measured volume of the dried slurry and the volume of the solid content in the dried slurry, respectively. The φ of the electrodes in the *half‐cells* is 65 % and 61 % for Cu‐MWGG‐1‐ and Al‐MWGG‐based electrodes, respectively, whereby a much lower φ was estimated for the commercial electrodes (30 % for the graphite electrode and 25 % for the LCO electrode). For the *full cells*, the φ was estimated to be 50 % as well as 39 % for the anodes and 50 % as well as 43 % for the cathodes for the MWGG‐based and the foil‐based electrodes, respectively, which is comparable.

In summary, it can be stated that the sheet resistance of the CCs has no significant influence on the rate capability here. The MWGG‐based electrodes are characterized by a higher resilience to higher C‐rates, which is suggested to be due to the larger CC surface area, shorter charge carrier path lengths, and the additional C coating on the Cu‐MWGG‐2 CC. However, the benefits from the higher φ can not be excluded.

#### Long‐Term Cycling

Three different types of *full cells* and three different cathode AMs (LCO, LiFePO_4_ (LFP), and LiNi_0.5_Mn_0.3_Co_0.2_O_2_ (NMC532)) were used to study and compare long‐term cycling behavior: cell‐type 1 with self‐coated foil‐based electrodes as a reference, cell‐type 2 with Cu‐MWGG‐1 CC (anode) and Al‐MWGG CC (cathode), and cell‐type 3 with Cu‐MWGG‐2 CC (anode) and Al‐MWGG CC (cathode). The *C*_g,both_ and the Coulombic efficiency are shown in Figure 7g, h for LCO and LFP, respectively, and in Figure S7 for NMC532. Compared to the foil‐based reference electrodes (cell 1, black), cell 2 (red) showed an increased *C*_g,both_ of about 26 % for LCO, 33 % for LFP, and 20 % for NMC532. For cell 3 (yellow), the increase in *C*_g,both_ is lower due to the heavier Cu‐MWGG‐2 CC (14 % for LCO, 6 % for LFP, and 12 % for NMC532) (see “Fabrication of MWGG CCs”). Besides, *C*_g_ of the MWGG‐based electrodes normalized to the electrode mass increased by 48 % and 14 % for the anode and cathode for LCO AM, respectively.

For the LCO cells, the difference in *C*_g,both_ is mainly due to the mass difference of the CCs, as they have comparable mass loading and ratio of negative to positive electrode capacity (*n*/*p* ratio) that is close to unity (see Table 1). Among the LFP cells, cell 2 also exhibited the highest *C*_g,both_ value but due to the combined effect of a lower CC area mass and a higher *n*/*p* ratio (1.3) of cell 1. In constrast, cell 3 has a lower initial capacity than cell 2, although the loadings for all the LFP cathodes (2.5 mA h cm^−2^) (Table S1) are identical, leading to a lower increase of *C*_g,both_ compared to cell 1. For NMC532 cells, the increases in *C*_g,both_ is generally lower for cell 2 and cell 3 (see Table S2), which might be due to their slightly higher *n*/*p* ratios (1.2).

**Table 1:** Experimentally determined parameters of the tested LCO cells. The parameters are the *n*/*p* ratio, the capacity *C*, the capacity *C*_g_ normalized to different cell components (anode/cathode=whole electrode mass; both=sum of both electrode masses; graphite/LCO=active material) after formation (“initial”) and after 300 cycles (“final”), the area capacity *C*_area, anode_, *C*_area, cathode_ (the geometrical area was used), the initial and averaged Coulombic efficiency *ICE* and <*CE*>, the modeled resistance values *R*_1_, *R*_2_, and *R*_3_, specific energy *e*, and energy density *u*. The best values for initial and final states are highlighted in bold, and “n.a.” stands for *not available*.

Cell	Cell 1		Cell 2		Cell 3	
	(foil‐CCs)		(MWGG CCs)		(MWGG CCs)	
Parameter	initial	final	initial	final	initial	final
*n*/*p* ratio	1.0		1.1		1.0	
*C*/mA h	4.2	3.2	**4.9**	**4.4**	4.3	4.0
*C*_g,anode_/mA h g^−1^	120	91	**177**	158	152	141
*C*_g,cathode_/mA h g^−1^	86	66	**98**	88	91	84
*C*_g,both_/mA h g^−1^	50	38	**63**	**57**	57	53
*C*_g,graphite_/mA h g^−1^	**267**	203	260	232	265	**246**
$C_{g,\;{\mathrm{LiCoO}}_{2}}$ /mA h g^−1^	111	85	**122**	**109**	114	105
*C*_area,anode_/mA h cm^−2^	2.6		3.1		2.6	
*C*_area,cathode_/mA h cm^−2^	3.0		3.2		3.0	
*ICE*/%	**92.7**		n.a.		82.8	
<*CE*>/%	99.9		100.0		**100.0**	
*R*_1_/Ω	2	2	2	2	**1**	**1**
*R*_2_/Ω	**2**	**2**	17	22	4	9
*R*_3_/Ω	89	38	59	30	**17**	**22**
*e*/W h kg^−1^	186		**232**		213	
*u*/W h L^−1^	**479**		436		411	

It shall be noted that an increased *C*_g_ brings an improvement in specific energy *e* (*e* = *UC*_g_, where *U* is the nominal cell voltage). The highest *e* are (232, 201, 263) W h kg^−1^ realized by LCO, LFP, NMC532 cell 2, respectively, which are (25, 33, 20)% higher than the foil‐based reference cells. It is important to note that the total mass of both electrodes was used for the calculation of *e*.

When it comes to the energy density *u*, we first estimate theoretical values and then calculate the experimental values obtained. For the theoretical values, we need the volume of the MWGG CCs which is the electrodes’ area and their thickness. We want to note that thickness here is from a virtual foil‐like MWGG CC. Since the area is known, we just need to determine the thickness of the MWGG CCs. This was already estimated within the aforementioned procedure of φ (Cu‐MWGG‐1: 9.7 μm, Cu‐MWGG‐2: 12.0 μm, and Al‐MWGG: 11.5 μm). Finally, we obtain a theoretical increase of 10 % and 40 % compared to the commercial Cu foil (9 μm thickness) for Cu‐MWGG‐1 and Cu‐MWGG‐2 CC, respectively, and a decrease of 27 % for the Al‐MWGG CC compared to the commercial Al foil (15 μm thickness). When we now take into account an identical slurry (composition and amount) compared to the commercial MTI electrodes on the MWGG CCs, the theoretical total thickness of both electrodes with Cu‐MWGG‐1/Al‐MWGG CCs and Cu‐MWGG‐2/Al‐MWGG CCs should decrease by 2.7 % and 0.5 %, respectively. This results in an increase of the theoretical volumetric capacity *C*_V,both_ and thus *u* of these combinations by 2.8 % and 0.5 %.

When it comes to the experimental results, the evaluation of *u* becomes more complex due to the interplay of the individual electrode parameters (thickness, φ , and mass loading), which are listed in Table S1. In general, the MWGG‐based electrodes have a higher φ in comparison to the foil‐based ones. However, for comparison, we selected electrodes with a comparable φ and mass loading. Hence, the anodes with Cu‐MWGG‐1 and Cu‐MWGG‐2 CCs as well as the cathode with Al‐MWGG CC showed a general increase in electrode thickness of 10 % and 20 % as well as 21 % in comparison to the foil‐based electrodes. This leads to a lowered *C*_V,both_ and *u* for the cells with MWGG‐based electrodes (*cf*. Table S2). In the case of LCO *full cells*, the *C*_V,both_ and *u* of cell 2 and cell 3 are decreased by 8 % and 9 % as well as 15 % and 14 %, respectively.

To summarize, contrary to the expected increase of *C*_V,both_ and *u* by (0.5–2.8) %, we obtained a decrease of at least 4 % for *C*_V,both_ and 3 % for *u*. We attribute this mainly to a remaining porosity component in all MWGG‐based electrodes. We would like to note that the total volume of both electrodes was used for the calculation of *u*.

In terms of cycling stability, cell 3 exhibited the best results for all the AMs, although its *C*_g,both_ is not the highest. The capacity retention for LCO cell 3 is 93 % after 300 cycles at 0.5 C, followed by cell 2 with 92 % and cell 1 with 76 %, which shows a high cycling stability of the MWGG‐based electrodes. LFP cell 2 and cell 3 exhibited a capacity retention after 300 cycles of 87 % and 90 %, respectively. The capacity behavior of LFP cell 1 has a different pattern during cycling (Figure 7h). We attribute this behavior to the decreased wettability due to the high calendering pressure applied on the electrodes^[47]^ (see Table S1). For NMC532, the capacity retention after 100 cycles for cell 2 and cell 3 are 84 % and 86 %, respectively, which are also higher than that of cell 1 (71 %).

We attribute this overall more stable behavior of the MWGG‐based cells to mainly two aspects: (*i*) MWGG CC flexibility and (*ii*) lower current density. It is expected that the flexibility of the MWGG CCs can mitigate the volume change of graphite during the intercalation/deintercalation process, leaving the SEI mostly intact with less crack formation. The continuous growth of SEI caused by volume change of the graphite AM is one of the capacity fading mechanisms in a LIB.^[48]^ We further attribute the overall better capacity retention of cell 3 to the fractal‐type morphology of the Cu‐MWGG‐2 CC, which provides better adhesion of the dried slurry mass on the CC and especially lower current densities. Besides, the additional C coating on the Cu‐MWGG‐2 CC provides protection against corrosion of the CC[^49^, ^50^] and should be another aspect that improves the cycling stability.

The Coulombic efficiencies in Figure 7g, h show that the cells with MWGG CCs perform similarly to the cells with foil‐CCs. The variances indicate that cell 2 is the most stable. However, side reactions cannot be excluded. The initial Coulombic efficiency (*ICE*), which characterizes the loss of Li in the first cycle, is comparable but lower for the cells with MWGG‐based electrodes. We assume that the higher interface area is the reason.

To characterize the impedance development of the cells, the potentio‐electrochemical impedance spectroscopy (PEIS) measurements were performed as a function of cycle number. A first measurement was performed after cell assembly and a rest time of 24 h (Figure S8). The Nyquist plot after the rest period only showed a comparably small semicircle, followed by a strong increase of the impedance. The other measurements (after formation, 100, 200, 300 cycles) were performed in the discharged state. The propagation of the impedances for LCO reference cell 1 (black), cell 2 (red) and cell 3 (yellow) with MWGG‐based electrodes is shown in Figure 7b, d, f, respectively. The Nyquist plots for the LFP cells are presented in Figure S9. In all these plots, the curves showed two semicircles with a subsequent linear increase. The curves are shifted to higher impedances with the increasing cycle number, indicating impedance growth due to battery degradation.

The Nyquist plots were modeled using the equivalent circuit of Ref. [51] (inset in Figure 7f). The modeled curves, resistances (*R*_1_, *R*_2_, and *R*_3_), and fitting errors (*χ* ^2^/|*Z*|) are shown in Figure 7b, d, f, Figure S9, Table 1, and Tables S3, S4, S5. *R*_1_ is the ohmic resistance known as bulk resistance, given from the left intersection of the data with the abscissa (Ref. [52]) in the Nyquist plot. *R*_2_ and *R*_3_ describe further contributions (see Ref. [52] for details).

In summary, cell 1 of all the AMs showed the highest impedances. The *R*_1_ values for all the cells are similar and stable during cycling (∼2 Ω). The behavior of *R*_2_ and *R*_3_ differed from cell to cell as the cycle number increased. However, cell 1 showed the highest *R*_2_ values for LFP and NMC532 and the highest *R*_3_ values for all the AMs. *R*_3_ is generally decreasing after formation, and about to gradually increase with the number of cycles. Thus, the cells with MWGG‐based electrodes are able to have comparable or lower impedance values than the reference foil‐based electrodes. The propagating pattern of the modeled resistances with the increasing cycle number for all the AMs remains complex.

In summary, the *full‐cell* cycling data (capacity and Coulombic efficiency) showed a similar cycling behavior for MWGG‐ and foil‐based electrodes but, as expected, higher *C*_g_ and *e* for the MWGG‐based electrodes. However, different from the predictions, the MWGG‐based electrodes have slightly decreased *C*_V,both_ and *u* values. A better capacity retention was found for the cells with the Cu‐MWGG‐2‐based anode with fractal‐type CC morphology. Lower impedances were generally observed for the cells with MWGG‐based electrodes.

## Conclusions

In conclusion, using coin‐cell format *full cells*, we have shown that novel MWGG CCs for future LIBs exhibit comparable properties and could have (with today′s LIB technology) remarkable benefits in terms of metal saving of up to 90 % and thus cost reduction of the same order for the CC metal costs, which preserves metal resources and reduces risks of raw material supply. This is the prerequisite for a more resource‐efficient LIB. At the same time, they allow for processing with existing production processes (using foil‐CCs). The woven‐glass‐grids can be converted to CCs and later electrodes in a R2R process using PVD methods, which enables production on large scales.

Moreover, utilizing the MWGG CCs improves gravimetric capacity (48 % and 14 % for the anode and cathode, respectively, in comparison to foil‐CCs) by reducing the mass of CCs by 60 % (anode side) and 28 % (cathode side). The overall mass can be further reduced by using thinner glass filaments, or yarns, by decreasing the thread density, or by reducing the thickness of the metal coating. The specific energy has thus been enhanced by 25 %. However, the energy density could not be increased to the expected values of maximal 2.8 % so far. A more flattened yarn and fewer filaments will further improve the value. Compared with self‐prepared foil‐based electrodes, the electrodes with Cu‐MWGG‐2 CC are further characterized by a slightly higher adhesion strength of the AM by 4 % (0.28 MPa) due to their specific fractal metal morphology with large surface area.

In our experiments, we also showed that the rate capability of the cells with MWGG‐based electrodes is higher, allowing for higher power density applications. We attribute this to three aspects: the higher surface area of the MWGG CC, and thus smaller current density, the embedding of the MWGG CCs into the active mass reducing the charge carrier travel path lengths by −7 % and −15 % for the anode and cathode, respectively, and the protective C coating. However, the effect of higher porosity should also be considered. Besides, the cells with MWGG‐based electrodes are able to show comparable or even lower impedances in comparison to the cells with foil‐based electrodes.

We could also demonstrate that the MWGG‐based electrodes exhibit good cycling stability with different cathode AMs (LCO, LFP, and NMC532) with, in particular, over 93 % and 90 % capacity retention after 300 cycles at 0.5 C for the LCO and the LFP system, respectively. This is up to 22 % higher in comparison to foil‐based electrodes. Besides, we also demonstrated that the MWGG CCs are stable within different chemical environments (LCO, LFP, NMC532). We attribute the better performance to improved mechanical flexibility, lower current density, better adhesion, and the protective C coating in the case of Cu‐MWGG‐2 CC.

Since glass can be recycled infinitely, these CCs also have great potential in terms of battery component recycling. Moreover, glass is a widely available raw material and has high mechanical and thermal stability as well.

For future development and improvement of LIB performance with MWGG CCs, every single detail in the manufacturing process is critical for reducing cost, increasing performance, and improving resource‐efficiency, *e. g*. slurry composition, porosity, charge carrier path length, internal/bulk resistance, overpotential of the electrodes, and surface area. This makes it difficult to finally assess the full potential of the MWGG CCs in LIBs and needs further investigation. In addition to improving the performance of LIBs at the coin cell level, the next step of this work will focus on scaling up LIBs with MWGG‐based electrodes to pouch cell and cylindrical cell formats and on investigating different types of MWGG CCs with a lighter glass substrate, *i. e*. increasing mesh size (free space; see an example in Figure S10) and decreasing the number of glass filaments. In the pouch cell format, electrode homogeneity, electrolyte wetting (benefit of MWGG CCs is expected), and contacting of the electrodes are critical for optimal electrochemical performance.

Finally, we were able to demonstrate a proof‐of‐concept for a lighter and more powerful LIB using metallized woven‐glass‐grid CCs, which we believe have great potential for future batteries.

## Experimental

### Fabrication of MWGG CCs

The here‐introduced CCs consist of up to three components: the support medium (woven‐glass‐grid), the functional metal (Cu or Al), and a protection layer (carbon). The support medium consists of flattened yarns composed of 104 e‐glass filaments of circular cross‐section and a diameter of 5 μm (EC5 5.5 tex; Agy Holding Corp.).^[53]^ The yarns have an intended oval linear shape to realize a flat electrode later and are interwoven to form a grid. In the warp and weft directions their widths are about 285 μm and 145 μm, respectively, while their thicknesses, that depend on the number of overlaying filaments, are ∼10 μm and ∼20 μm, respectively. The different thicknesses of the warp and weft yarns are due to the different mechanical loads on the yarns when the woven‐grid is transported through the winding system during the weaving process. The woven‐glass‐grid has a final cross‐sectional extent of (30–33) μm on this basis. We refer to *cross‐sectional extent* rather than thickness because the woven‐glass‐grid is only about 30 μm thick in the nodal regions where the warp and weft yarns are crossing. The yarn (thread) density is 22 warp and 22 weft yarns (threads) per cm.

These woven‐glass‐grids, that are reliably available, were then processed into CCs by elfolion GmbH using dedicated patented process technologies^[15]^ in a continuous R2R process (Figure S11). The core of the processing is the metallization of the woven‐glass‐grids by enveloping deposition of the functional materials that provide electrical conductivity (Cu or Al) by using various sequential PVD processes. This circumvents a wet‐chemical approach as described in Ref. [9]. The metal deposition can be carried out in such a way that fractal solid‐state structures are realized. The amount of metal applied corresponds to a metal foil with a thickness of 1 μm. A final carbon anti‐corrosion layer can be applied. These processed woven‐glass‐grids are referred to as *metallized woven‐glass‐grid* CCs.

For the preparation of electrodes, we used the following MWGG CCs:

– Cu‐MWGG‐1 (Cu coating, area density: 32 g m^−2^),
– Cu‐MWGG‐2 (fractal Cu coating, fractal C finish, area density: 50 g m^−2^), and
– Al‐MWGG (Al coating, area density: 30 g m^−2^).

Characterizing parameters of the MWGG CCs are summarized in Table S6.

A qualitative and quantitative phase analysis of the MWGG CCs were performed by transmission powder XRD using a D8 Discover diffractometer (Bruker AXS) equipped with a Goebel mirror tuned to the Cu‐*Kα*‐radiation (*λ*_Cu‐*Kα*_ = 1.5419 Å), and a LYNXEYE XE‐T detector in 1D mode. The diffraction patterns were recorded in the range of 2*θ* = (10–110)°, with a step width Δ2*θ* = 0.02°, and a collection time per step of *t* = 1 s at ambient conditions. The recorded data were analyzed qualitatively using the program EVA (Bruker AXS) and the Powder Diffraction File^[54]^ and quantitatively by Rietveld refinement using the program TOPAS V6.^[55]^

The surface composition of the MWGG CCs was characterized by XPS using an ESCALAB 250Xi (Thermo Fisher Scientific) spectrometer, which excites the sample by monochromatic Al‐*Kα* radiation (spot diameter: 650 μm) and uses a MAGCIS Ar cluster ion sputtering source for depth profiles (duration of >540 s). To calibrate the binding energy values, we set the main C 1s peak to 284.8 eV.

The morphology and microstructure of the MWGG CCs and later electrodes were characterized by SEM using a Helios NanoLab 600i (FEI) at an acceleration voltage of 10 kV and by confocal microscopy using a LEXT 3D laser scanning microscope OLS4100 (Olympus). The latter was especially used for microstructural characterisation, *i. e*. surface waviness and surface roughness. For the analysis a tilting of the height profiles was corrected, a noise correction for stepped surfaces was carried out, and a cut‐off wavelength of 100 μm and 500 μm for waviness and roughness, respectively, was chosen.

The surface area of the CCs was measured by BET analysis^[56]^ using an SA3100 (Beckman Coulter GmbH) instrument. The samples were stored in glass tubes, degassed at a temperature of 200 °C for 2 h and analyzed under nitrogen atmosphere (adsorption‐desorption isotherms at 45 °C).

The electrical sheet resistance was measured by linear 4‐point probe measurement. A self‐constructed 4‐point probe measuring tool from elfolion GmbH was used with equal spacing between test electrodes with a diameter of 12 mm for contact. The current was set 10 mA in the measurement.

Mechanical characterization of different samples (woven‐glass‐grid base material, MWGG CCs, Al/Cu foil (Xiamen TOB New Energy Technology); strip width/length: 50 mm/200 mm) along the fabric length (weft direction) was carried out by strip tensile tests[^18^, ^57^] according to DIN EN ISO 13934‐1^[58]^ using a Z2.5 (ZwickRoell GmbH & Co. KG) tensile testing machine. For each sample, 2–4 tests were performed.

### Electrode Preparation, Cell Assembling, Electrochemical and Adhesion Tests

The electrodes were prepared by doctor blading the slurries. For the anode slurry, mesocarbon microbeads (MCMB) graphite powder (Xiamen TOB New Energy Technology; particle size (D90): 18 μm) was used as AM, carbon black powder (Alfa Aesar, CAS‐Nr.: 1333–86‐4) as conductive additive, and carboxymethyl cellulose (CMC) powder (Xiamen TOB New Energy Technology) and styrene‐butadiene rubber (SBR) (Xiamen TOB New Energy Technology) as binders (1 : 1). They were mixed in a 90 : 5 : 5 ratio (AM : conductive additive : binder) using a dissolver (IKA T25 Easy Clean Digital ULTRA‐TURRAX) at rotations of 12,000 min^−1^. Distilled water was used as the solvent, and the mass fraction of the solid content was 40 %, resulting in the zero shear viscosity of 14.0(5) Pa s for the anode slurry.

For the cathode slurry, LCO powder (Xiamen TOB New Energy Technology; particle size (D90): 16.722 μm), LFP powder (Xiamen TOB New Energy Technology; particle size (D90): <10 μm), and NMC532 powder (Xiamen TOB New Energy Technology; particle size (D90): <25.0 μm) as AM, carbon black powder as conductive additive, and polyvinylidene fluoride (PVDF) powder (Sigma–Aldrich, CAS‐Nr.: 24937–79‐9) as binder were mixed in the same ratio and way as for the anode. *N*‐Methyl‐2‐pyrrolidone (NMP; Sigma‐Aldrich, CAS‐Nr.: 872–50‐4) was used as the solvent while the mass fraction of the solid content was 50 %, resulting in an LCO slurry with the zero shear viscosity of 1603(137) Pa s.

We determined the viscosity of the slurries by the rotary rheometer RHEOTEST RN 4.1 (RHEOTEST Medingen GmbH). We applied <0.3 mL of the slurry onto the K3 cone‐plate measuring system, and the gap between the cone and the plate was set to the standard value of 40 μm. The actual measurement was performed with an increasing shear rate (from 0.1 s^−1^ to 1000 s^−1^ within 2 min) and the Cross model was utilized for analysis. We want to note that the errors of the zero shear viscosities were calculated by the maximum difference of measured and averaged values because the modeling precision did not account for the observed differences that are likely due to slurry inhomogeneities.

In addition, pure Li foils with the thickness of 0.38 mm (Sigma‐Aldrich, CAS‐Nr.: 7439‐93‐2) were used to assemble *half cells* whereas commercial electrodes (anode: BC‐CF‐241‐SS, MTI Corp; cathode: TOB‐Electrode‐LiCoO_2_, Xiamen TOB New Energy Technology) were used for comparison.

For further comparison purposes and for rating the novel MWGG CCs, Cu and Al foil‐CCs were coated and processed with the same slurry and procedure as the MWGG CCs.

The MWGG CCs (Cu‐MWGG CC was used for the anode, and Al‐MWGG CC for the cathode) were attached to a polytetrafluoroethylene foil. The slurry was then applied with a coater (Xiamen TOB New Energy Technology) on top of the CCs. The obtained mass loading of each electrode is given in Table S1. The electrodes were dried in a furnace (Memmert UNB 100 Oven) at a temperature of 80 °C for 12 h at ambient air. Anode and cathode discs with diameters of 16 mm and 15 mm were then cut using an electrode cutter (TOB‐CP60, Xiamen TOB New Energy Technology) and finally calendered vertically by application of a pressure of (25–83) MPa (Table S1) using a mechanical press (MP150, Maassen GmbH).

For the assembly of CR2016 coin cells (Xiamen TOB New Energy Technology) under inert atmosphere (glovebox, Sylatech GmbH), we used commercially available electrolyte (1 M LiPF_6_ salt mixed with DEC and EC at a volume ratio of 1 : 1; Sigma–Aldrich, PCode: 1003231860) and Celgard 2325 as separator (diameter of 19 mm). The cells were pressed with a pressure of 50 kg cm^−2^ in a coin cell press (MSK‐110, MTI Corp).

The battery cycler BCS‐810 (BioLogic SAS) was used for the electrochemical tests: LSV, CV, galvanostatic cycling with potential limitation (GCPL), and PEIS.

The LSV of uncoated CCs (MWGG CCs, Cu and Al foil) was conducted at a scan rate of 0.5 mV s^−1^ and 1 mV s^−1^ from open‐circuit potentials to −0.24 V and 6.5 V vs Li+/Li for Cu and Al, respectively, with Li metal as reference electrode and using the same electrolyte as above. For calculating current densities the geometrical area of the electrodes was used.

The CV of electrodes was performed at a scan rate of 0.1 mV s^−1^ between a voltage of 0.01 V and 3 V for the graphite, and between 3 V and 4.2 V vs Li+/Li for the LCO electrode with Li metal as the reference electrode.

Regarding the cycling procedure, we monitored the coin cells with open circuit voltage for 24 h after assembly to provide enough time for reaching equilibrium among the cell components (wetting, soaking of electrolyte) before performing the first PEIS measurement (frequency range: 10 mHz to 10 kHz, voltage amplitude: 10 mV, averaging: two measurements per frequency, recording 10 points per decade). An evaluation of the errors for the impedances by measuring the same state several times resulted in deviations in the order of 2 Ω.

Next, a formation step was done by GCPL at 0.1 C (0.01 A g^−1^) in a voltage range of 3.0 V < *E*_Cell_ < 4.2 V for LCO cells, 2.5 V <*E*_Cell_ < 3.8 V for LFP cells, and 2.75 V <*E*_Cell_ < 4.3 V for NMC532 cells for up to 5 cycles to form the SEI. Then, for the electrochemical testing, either several series of PEIS and GCPL at 0.5 C (0.07 A g^−1^) for 100 cycles were performed to investigate the cyclability, or a series of GCPL at different C‐rates was carried out to test the rate capability. All procedures were performed at ambient conditions. The series of C‐rates were 0.1 C (0.01 A g^−1^), 0.25 C (0.03 A g^−1^), 0.5 C (0.07 A g^−1^), 1 C (0.14 A g^−1^), 2 C (0.28 A g^−1^), 3 C (0.4 A g^−1^), 0.25 C (0.03 A g^−1^), and at each C‐rate, the cells were cycled at least 5 times. Typical cycle curves for the LCO *full cells* are shown in Figure S12.

A mechanical adhesion test with the electrodes was performed using a BZ2.5/TS1S tensile tester (ZwickRoell GmbH & Co. KG) equipped with a self‐constructed stainless steel specimen holder Figure S5. We followed the adhesion test procedure of Ref. [35]. Four electrodes for each CC were first cut into 12 mm‐diameter discs identical to the specimen holder. Adhesive tape (tesa BNR 5696) was attached to the specimen holder′s surfaces so that both surfaces of the tested electrode could come into contact with the tape to separate the dried slurry and CC. The maximum contact force was set to 68 N, corresponding to a pressure of 600 kPa. After a holding time of 30 s under the maximum contact force, the upper column was pulled upwards and the adhesion force was recorded (Figure S5). Subsequently, the adhesion strength of the electrodes was calculated from the maximum pull‐off force divided by the loaded area (here the geometrical area) according to Ref. [35].

## Supporting Information

The authors have cited additional references in the Supporting Information.[^59^, ^60^]

## Conflict of Interests

Yen‐Ming Li, Winfried Voitus, and Manfred Danziger are working for elfolion GmbH.

1

## Supporting information

As a service to our authors and readers, this journal provides supporting information supplied by the authors. Such materials are peer reviewed and may be re‐organized for online delivery, but are not copy‐edited or typeset. Technical support issues arising from supporting information (other than missing files) should be addressed to the authors.

Supporting Information

## Data Availability

The data that support the findings of this study are available from the corresponding author upon reasonable request.
