# Supplementary material for: Resource‐Efficient Electrodes with Metallized Woven‐Glass‐Grid Current Collectors for Lithium‐Ion Batteries
Source: ChemSusChem. 2024 Dec 3;18(6):e202402233. doi: 10.1002/cssc.202402233 (PMC11911995; doi:10.1002/cssc.202402233)
Supplement: Supplementary file 1 — Supporting Information [file CSSC-18-e202402233-s001.pdf]

ChemSusChem

Supporting Information

Resource-Efficient Electrodes with Metallized Woven-Glass-Grid Current Collectors for Lithium-Ion Batteries

Yen-Ming Li,* Mohammadjafar Momeni, Huy Nguyen Dang Duc, Suvi von Bahder, Friedrich Roth, Wolfram Münchgesang, Manfred Danziger, Winfried Voitus, Dominik Nuss, Cornelia Sennewald, and Tilmann Leisegang*

Supporting Information

Resource-efficient electrodes with metallized woven-glass-grid current collectors for lithium-ion batteries

Yen-Ming Li[a,b,*], Mohammadjafar Momeni[a], Huy Nguyen Dang Duc[a], Suvi von Bahder[a], Friedrich Roth[a,c], Wolfram Münchgesang[d], Manfred Danziger[b], Winfried Voitus[b], Dominik Nuss[e], Cornelia Sennewald[e], Tilmann Leisegang[a,*]

[a]Institute of Experimental Physics, TU Bergakademie Freiberg, Leipziger Str. 23, Freiberg, 09599, Saxony, Germany

[b]elfolion GmbH, Magdeburger Str. 7, Quedlinburg, 06484, Saxony-Anhalt, Germany

[c]Center for Efficient High Temperature Processes and Materials Conversion (ZeHS), TU Bergakademie Freiberg, Winklerstrase 5, Freiberg, 09599, Saxony, Germany

[d]Institute for Wind Energy Systems, Fraunhofer, Großer Westring 2, Bremerhaven, 27572, Bremen, Germany

[e]Institute of Texile Machinery and High Performance Material Technology, TU Dresden, Hohe Str. 6, Dresden, 01069, Saxony, Germany

Table of Contents

	Page
Figure S1	3
Note S1	3
Figure S2	4
Note S2	4
Figure S3	5
Note S3	5
Figure S4	6
Figure S5	6
Figure S6	7
Figure S7	7
Figure S8	8
Figure S9	8
Table S1	9
Table S2	10
Table S3	11
Table S4	11
Table S5	11
Figure S10	12
Figure S11	12
Table S6	13
Figure S12	13

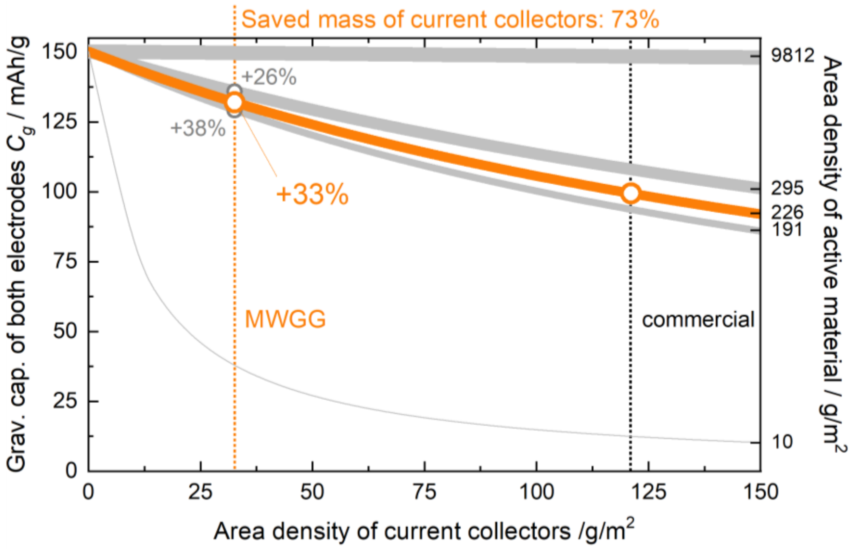

Figure S1: Estimation of the increase in theoretical gravimetric capacity C_g normalized to the AM of both electrodes. The gain in C_g depends on both, the area density of the CCs and the amount of AM used. The line thickness indicates the AM quantity (thicker = more), with the orange line representing the AM quantity used in commercially available electrodes (MTI Corp).

Figure S1 shows the estimated benefit of $C_{g,\ both}$ when using the MWGG CCs presented here. Accordingly, an increase of 33%, *i.e.* a factor of 1.33, can be expected.

Note S1: Calculation of the specific energy for a Tesla Model 3 battery with MWGG-CCs

We calculated the specific energy *e* for a Tesla Model 3 battery with MWGG-CCs as follows:

$$e = \frac{E_0}{m},$$

where the energy E_0 = 100 kWh [1] and the battery mass is

$$m = m_{CC} + m_{rest},$$

where m_{CC} and m_{rest} are the masses of the CCs and the battery without CCs. If we change the CCs for such battery we need to determine the mass of the used foil CCs, which we do with the help of Figure 1, that is $m_{CC,\ foil} = 0.15m$, which is 15% of the whole battery mass. Substituting the foil CCs then by MWGG ones with the help of Figure S1 then results in $m_{CC,\ MWGG} = 0.27m_{CC,\ foil}$, which is 27% of the mass of the foil CCs.

The energy gain can then be estimated by multiplying *e* of the Tesla Model 3 battery with the mass *m* = 355 kg [2] and the reduced mass due to substitution of the CCs. The increase in range was finally calculated based on the energy requirement per 100 km (15.87 kWh [1]) and the estimated energy gain.

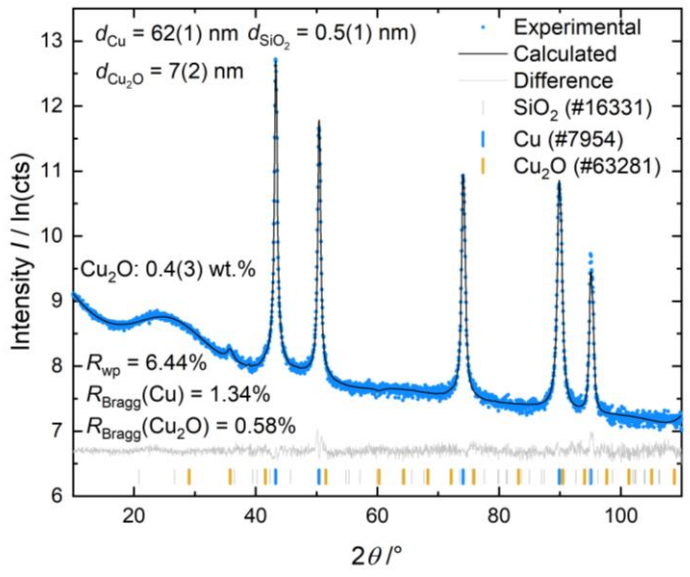

Figure S2: X-ray diffraction pattern of the Cu-MWGG-2 CC. Note the logarithmic scaling of the ordinate. A Cu_2O phase was observed with the most intense reflection at around 2θ = 35° and an estimated weight share of <0.5%.

Figure S2 shows the estimated Cu_2O content for the Cu-MWGG-2 CC, which has the highest amount of Cu and the highest surface area due to the fractal morphology of the metal coating.

Note S2: Invisibility of the C coating by X-ray diffraction.

We would like to point out that the intensities in Figure 3a, b and S2 are shown in a logarithmic scaling of the ordinate. This emphasizes particularly low intensities. From this, we cannot conclude any additional reflection to the ones already identified. This is also indicated by the Rietveld refinement, which led to low quality indicators (*R* values) that give no reason for an unobserved phase. Due to the low scattering ability of C compared to Cu (6 vs. 29 electrons) or Si (14 electrons) of the glass grid and due to the background intensity of the amorphous glass and the background radiation due to the irregular CC morphology, all this contributes to the invisibility of C. Therefore, neither an additional crystalline nor an amorphous phase can be recognized in the diffraction pattern of the Cu-MWGG CC (Figure S2).

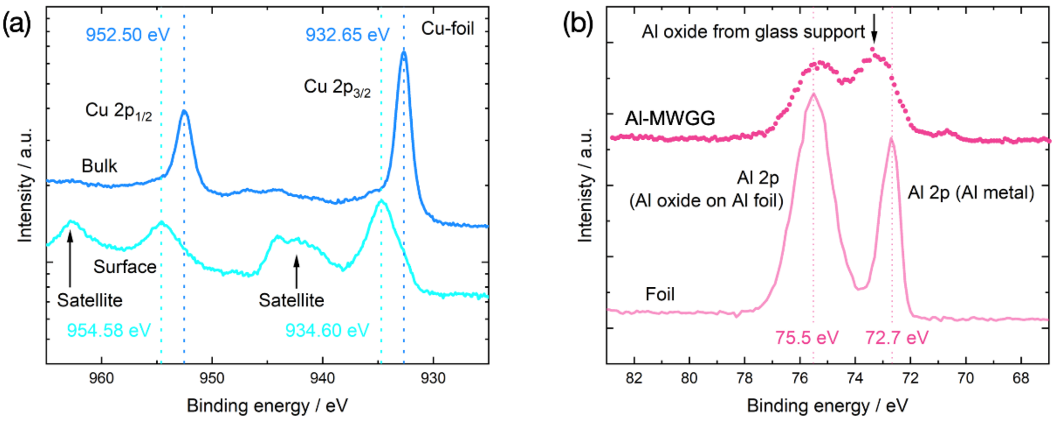

Figure S3: XPS data of (a) before (turquoise) and after (blue) sputtering the surface of Cu foil and of (b) the Al-MWGG CC (pink) and the Al foil (light pink). In (a) the Cu $2p_{1/2}$ and Cu $2p_{3/2}$ are indicated, while in (b) the Al 2p for Al oxide and Al metal can be seen.

The details of Figure S3a can be found in the manuscript ("Microstructure, morphology, and mechanical stability of the CC"). In Figure S3b the Al foil and the Al-MWGG CC is compared. We can see similarities between both. However, the peak widths are larger for the Al-MWGG CC, which we attribute to extrinsic broadening from sample charging effects. Additionally, the higher relative intensity of the peak at around 73.3 eV is attributed to an Al oxide component from the e-glass *support*.

Note S3: Fractality of the Cu metal coating on Cu-MWGG-2 CC

We want to emphasize that our vacuum-engineered solid-state structures (Figure 4i, k, m) are highly porous structures with a large surface area. With "fractality" we emphasize the additive manufacturing and characterize the morphology of the solid-state structures. Fractal solid-state structures can be created using a dedicated vacuum coating process, such as the one we used here. This process belongs to the additive manufacturing processes. They are based on the method of building up a structure or a morphology layer by layer from increments of a material (*e. g.* droplets), in this case Cu or Al. If looking at these structures with a scanning electron microscope at different magnifications, one can see self-similar structures as well as a high degree of branching, both being characteristics of a fractal nature. It should be noted that experimental fractal structures are characterized by a certain degree of randomness and are not as strict as mathematical objects. With these characteristics that our solid-state structures fulfill – (*i*) additive manufacturing, (*ii*) repetitive structural motifs / self-similarity, (*iii*) high degree of branching – we therefore consider the term "fractal" to be justified. References [3, 4] for example describe the characteristics of fractal structures and show examples for metals, also similar to the ones presented here. We are going to address the fractality and fractal dimension of the coatings by SEM, TEM, and image processing software elsewhere.

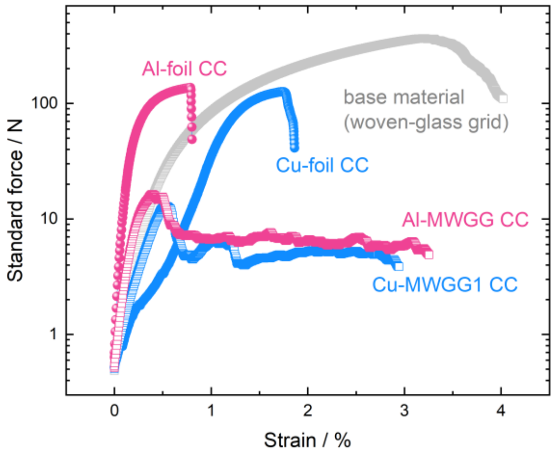

Figure S4: Tensile test results represented as standard force in dependence on strain obtained from the stripe tensile test.

Figure S4 shows the same data as Figure 5b, but in terms of standard force instead of tensile stress as the ordinate. In fact, this is the better representation of the data in the textile sector, as it gives an insight into the handling of the material. From this it can be concluded that the woven glass grids (base material) can be processed in the same R2R systems as the foil CCs. However, when processing the MWGG CCs, a transportation force in the order of 3 N (at the moment) should not be overcome in the R2R process.

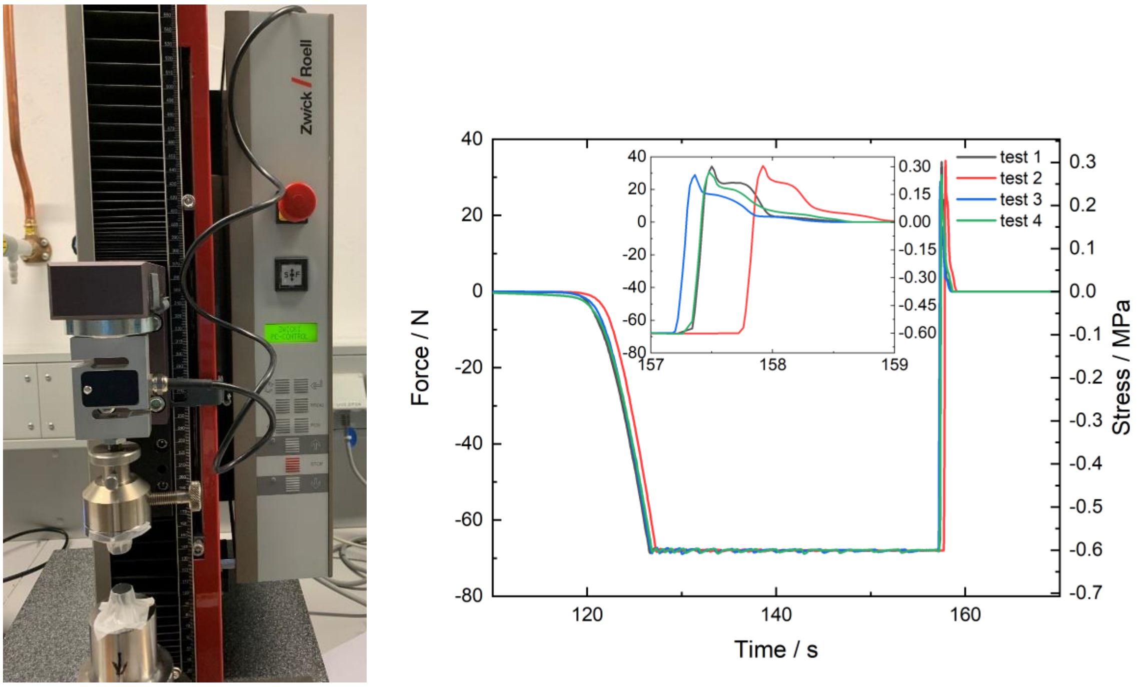

(a) (b)

Figure S5: (a) The adhesion strength testing setup of the tensile tester (BZ2.5/TS1S, Zwick/Roell) and its sample holders with tape and a sample (lower left). (b) An example of raw curves for the mechanical adhesion test of electrodes with Cu-MWGG-2 CC. The inset shows the maximum force obtained from each test. Details are described in “Electrode preparation, cell assembling, and electrochemical and adhesion tests”.

Figure S5 shows the adhesion test results of the four tested graphite electrodes with Cu-MWGG-2 CC. The general shape of all curves is similar, but the maximum (adhesion) force is different.

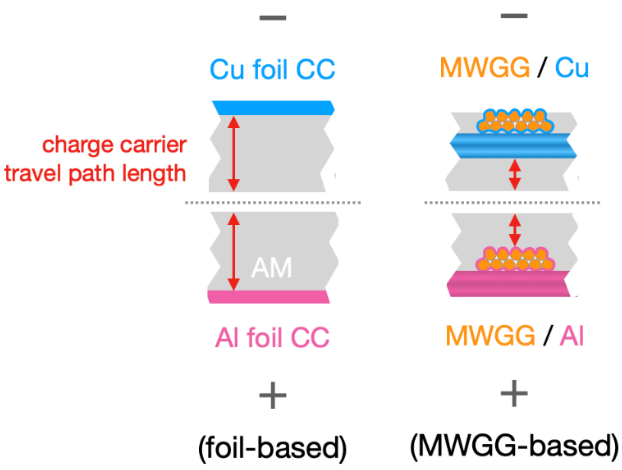

Figure S6: Charge carrier path lengths of foil- and MWGG-based electrodes.

Figure S6 schematically show the charge carrier path lengths for the foil- and MWGG-based electrodes. The distance from the electrode surface (separator side) to the interface of the dried slurry and the CC is considered the *charge carrier path length*. For the MWGG CCs, the shortest path length is located at the nodes of the grid. We estimated the path lengths based on the parameters of the used electrodes in the full cells for the rate capability test (Figure 7e): 74.7 µm and 69.7 µm for the Cu-foil-based and the Cu-MWGG-2-based anodes, and 77.3 µm and 66.0 µm for the Al-foil-based and Al-MWGG-based cathodes, respectively. Therefore, utilizing MWGG CCs leads to a charge carrier path length reduction by 7% for the anode and 15% for the cathode.

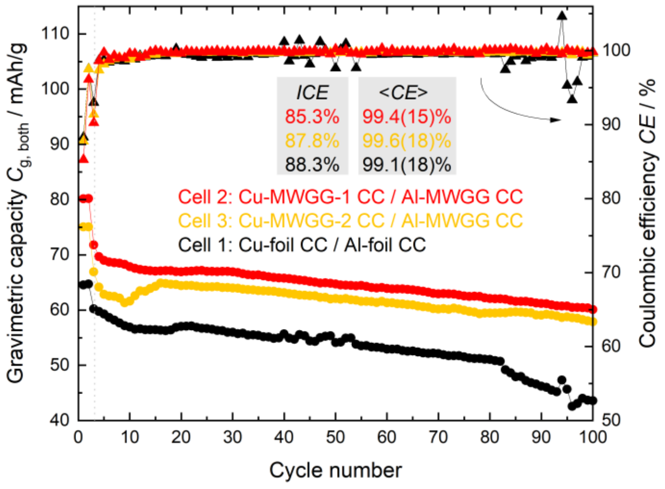

Figure S7: Cycling data of the cells with NMC532 AM used for the cathodes. Shown are the gravimetric capacity normalized to both electrode masses and the Coulombic efficiencies for the reference cell 1 (self-coated foils; black) and the cells with MWGG-based electrodes, cell 2 with Cu-MWGG-1 CC (red) and cell 3 with CU-MWGG-2 CC (yellow). The same coating and assembling procedures as for the other cells (Figure 7g, h) were used.

Figure S7 summarizes the cycling data of the different types of cell with NMC532 as AM for the cathode. Details can be found in the main article. It has to be noted that only 100 cycles were performed to show the general feasibility.

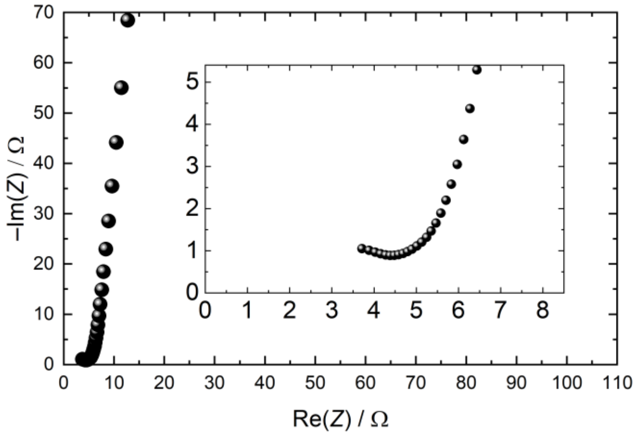

Figure S8: An example Nyquist plot for cell 1 (foil electrodes) with LCO AM recorded after assembling and a rest period of 24 h before cell cycling. The inset shows the enlarged low impedance region.

Figure S8 shows an example PEIS curve in a Nyquist plot for cell 1 with LCO AM. A similar characteristic is found for the corresponding cell 2 and cell 3. Details can be found in the main article in section "Long-term cycling".

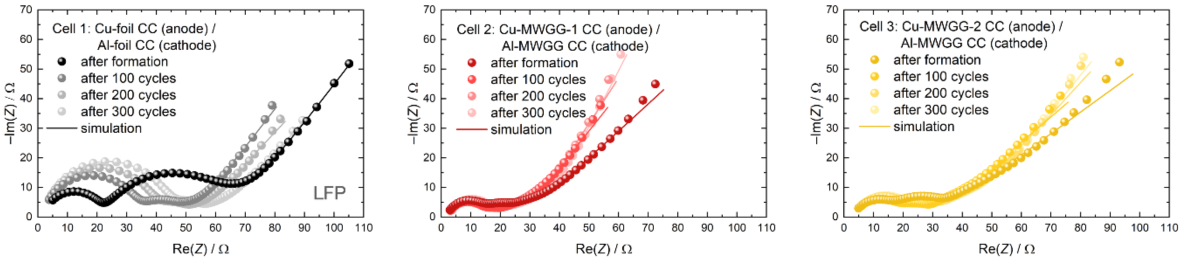

Figure S9: The Nyquist plots after formation, 100, 200, and 300 cycles for three different LFP-based cell-types: cell 1 with foil-based CCs (black), cell 2 with the Cu-MWGG-1 CC (red), and cell 3 with the Cu-MWGG-2 CC (yellow).

Figure S9 shows the PEIS data (Nyquist plots) for the different cell types with LFP AM for the cathode. Details can be found in the main article.

Table S1: Listed are the thickness, the porosity, the area loading, and the calendering pressure for the anode and cathode for the LCO, LFP, and NMC532 full cells, respectively.

Cell	Electrodes	Thickness / µm		Porosity / %		Loading / $mAhcm^{-2}$		Calendering pressure / MPa	
		Anode	Cathode	Anode	Cathode	Anode	Cathode	Anode	Cathode
**LCO**									
1	Cu foil/Al foil	81	90	45	32	2.6	3.0	25	31
2	Cu-MWGG-1/ Al-MWGG	105	113	48	45	3.1	3.2	30	36
3	Cu-MWGG-2/ Al-MWGG	97	109	51	47	2.6	3.0	31	40
**LFP**									
1	Cu foil/Al foil	84	105	41	43	2.8	2.5	73	83
2	Cu-MWGG-1/ Al-MWGG	82	127	50	55	2.2	2.5	33	39
3	Cu-MWGG-2/ Al-MWGG	95	129	54	55	2.4	2.5	32	39
**NMC 532**									
1	Cu foil/Al foil	72	95	40	53	2.4	2.5	37	42
2	Cu-MWGG-1/ Al-MWGG	92	110	40	50	3.0	2.9	37	42
3	Cu-MWGG-2/ Al-MWGG	99	103	46	49	3.1	3.0	37	42

Table S2: Listed are the experimentally obtained values of the gravimetric capacity $C_{g, both}$, the volumetric capacity $C_{V, both}$, the specific energy *e*, and the energy density *u* for the LCO, LFP, and NMC532 full cells, respectively. The relative differences of the values with respect to cell 1 are indicated.

Cell	Electrodes	$C_{g, both}$ / mAhg^{-1}		$C_{V, both}$ / mAhcm^{-3}		*e* / Whkg^{-1}		*u* / WhL^{-1}	
**LCO**									
1	Cu foil/Al foil	50		129		186		479	
2	Cu-MWGG-1/ Al-MWGG	63	+26%	119	–8%	232	+25%	436	–9%
3	Cu-MWGG-2/ Al-MWGG	57	+14%	110	–15%	213	+15%	411	–14%
**LFP** 1	Cu foil/Al foil	49		103		150		316	
2	Cu-MWGG-1/ Al-MWGG	65	+33%	99	–4%	201	+33%	305	–3%
3	Cu-MWGG-2/ Al-MWGG	52	+6%	79	–23%	158	+5%	242	–23%
**NMC 532** 1	Cu foil/Al foil	60		136		220		497	
2	Cu-MWGG-1/ Al-MWGG	72	+20%	129	–5%	263	+20%	473	–5%
3	Cu-MWGG-2/ Al-MWGG	67	+12%	128	–6%	243	+10%	464	–7%

Table S3: Modeled resistance values (R_1, R_2, R_3) and the error of fitting ($\chi^2/|Z|$) of the cells with LCO cathodes at different cycling states: after formation and after 100, 200, and 300 cycles.

**Cell**	**Cell 1: Cu foil/Al foil**	**Cell 2: Cu-MWGG-1/Al-MWGG**	**Cell 3: Cu-MWGG-2/Al-MWGG**
R_1/Ω	2, 2, 2, 2	2, 2, 2, 2	1, 1, 1, 1
R_2/Ω	2, 4, 3, 2	17, 17, 20, 22	4, 5, 6, 9
R_3/Ω	89, 36, 47, 38	59, 26, 24, 30	17, 13, 16, 22
$\chi^2/|Z|/\%$	0.3, 1.2, 0.3, 0.5	1.7, 0.1, 0.1, 0.2	1.8, 0.7, 0.7, 1.2

Table S4: Modeled resistance values (R_1, R_2, R_3) and the error of fitting ($\chi^2/|Z|$) of the cells with LFP cathodes at different cycling states: after formation and after 100, 200, 300 cycles. We used the same equivalent circuit model as for the cells with LCO cathode AM.

**Cell**	**Cell 1: Cu foil/Al foil**	**Cell 2: Cu-MWGG-1/Al-MWGG**	**Cell 3: Cu-MWGG-2/Al-MWGG**
R_1/Ω	2, 2, 2, 2	2, 2, 2, 2	2, 3, 3, 3
R_2/Ω	18, 30, 35, 37	12, 11, 12, 12	14, 17, 23, 22
R_3/Ω	47, 21, 17, 23	15, 17, 19, 28	15, 12, 16, 20
$\chi^2/|Z|/\%$	0.8, 0.1, 0,2, 0.1	1.0, 0.9, 1.0, 0.3	1.5, 1.0, 1.1, 0.9

Table S5: Modeled resistance values (R_1, R_2, R_3) and the error of fitting ($\chi^2/|Z|$) of the cells with NMC532 cathodes at different cycling states: after formation and after 100 cycles. We used the same equivalent circuit model as for the cells with LCO cathode AM.

**Cell**	**Cell 1: Cu foil/Al foil**	**Cell 2: Cu-MWGG-1/Al-MWGG**	**Cell 3: Cu-MWGG-2/Al-MWGG**
R_1/Ω	3, 3	1, 2	2, 3
R_2/Ω	14, 10	10, 7	10, 9
R_3/Ω	34, 28	23, 24	26, 20
$\chi^2/|Z|/\%$	8.4, 7.3	1.6, 1.2	3.3, 1.7

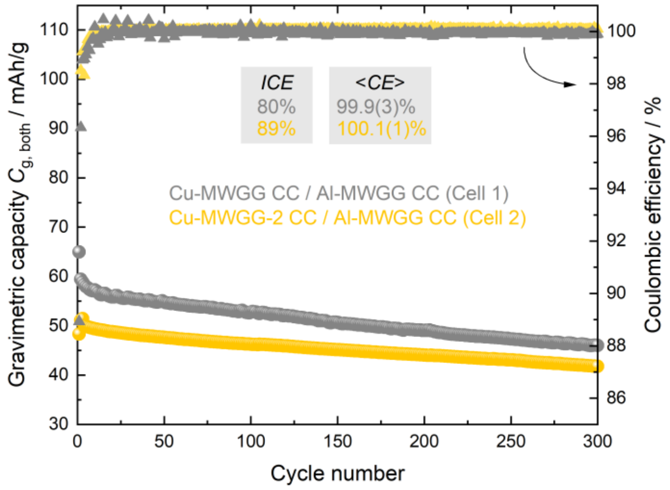

Figure S10: Gravimetric capacity normalized to both electrode masses and the Coulombic efficiencies of LFP full cells with different types of Cu-MWGG CCs. Cell 1 (gray) comprises a Cu-MWGG CC with a larger mesh size while cell 2(yellow) utilizes the Cu-MWGG-2 CC.

Figure S10 shows the potential of a lighter (less threads) Cu-MWGG CC. Here, the Cu-MWGG CC with a larger mesh size, which leads to a 61% increase in “pore area”, can be shown to perform comparably to the reported heavier Cu-MWGG-2-CC. Due to the 66% reduction in the areal mass of the Cu-MWGG (cell 1, gray), the $C_{g,\text{ both}}$ value is also higher here.

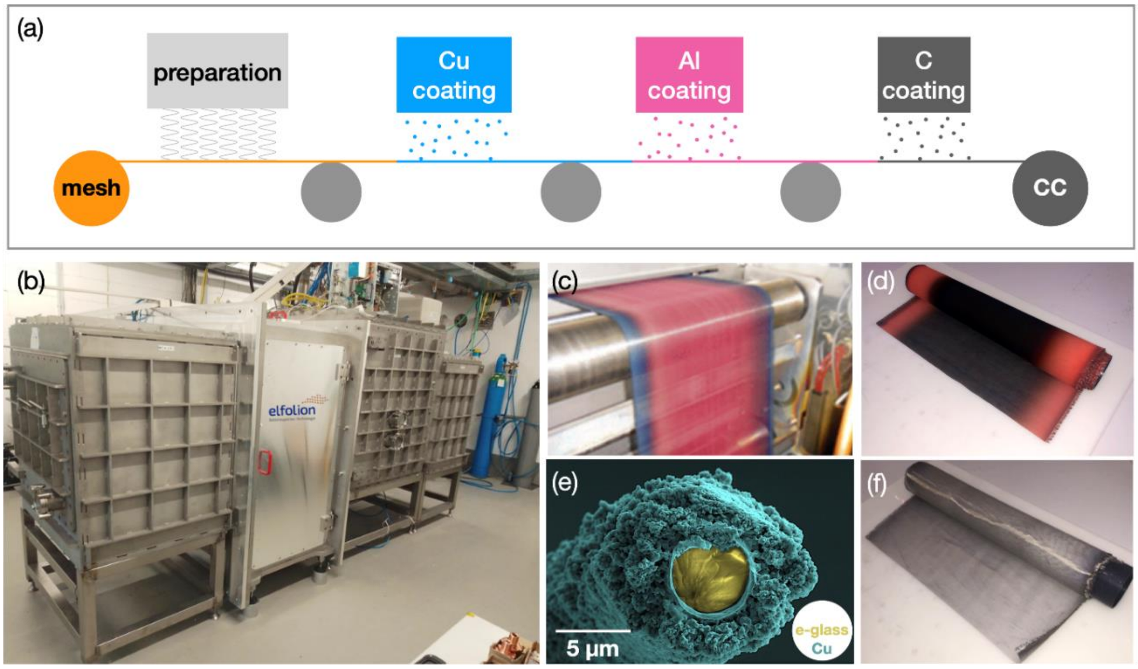

Figure S11: Processing of the woven-glass-grids to CCs using dedicated patented process technologies in a continuous R2R process (elfolion GmbH): (a) process scheme, (b) PVD coating system, (c, d) processed Cu-MWGG CC, (e) false-color SEM image of one single glass filament (yellow) enveloped by Cu (turquoise) demonstrating the metallization (note that actually several filaments are enveloped by one metal layer), (f) Al-MWGG CC.

In Figure S11 the processing of the woven glass grid to MWGG CCs is shown (a). The prototype PVD system is shown in (b) with which the woven-glass-grid-coating to CCs (c) can be carried out. (d) and (f) show prototypes of the Cu-MWGG-2 and Al-MWGG CC. (e) shows an example of a coated single e-glass filament.

Table S6: Listed are the characteristics of the investigated CCs: total area weight, area weight of Al/Cu coating, maximum expansion/thickness, composition, and supplier. Note that the mass of the commercial Al foil is calculated from the given thickness, and the mass of the Cu foil is given by MTI Corp.

CC	Type	Area weight / gm^{-2}		Thickness /µm	Composition	Supplier
		CC	Al/Cu			
Al-MWGG	basic	30.0 ± 0.8	5.47	37 ± 5	e-glass/Al	elfolion GmbH
Al foil		43.2	–	16	Al	Xiamen TOB New Energy
Cu-MWGG-1	basic	32.0 ± 1.3	9.575	34 ± 7	e-glass/Cu	elfolion GmbH
Cu-MWGG-2	Fractal, C-finish	50.4 ± 2.0	26.49	45 ± 4	e-glass/Cu/C	elfolion GmbH
Cu foil	bc-cf-241-ss-005	80.46	–	9	Cu	MTI Corp.

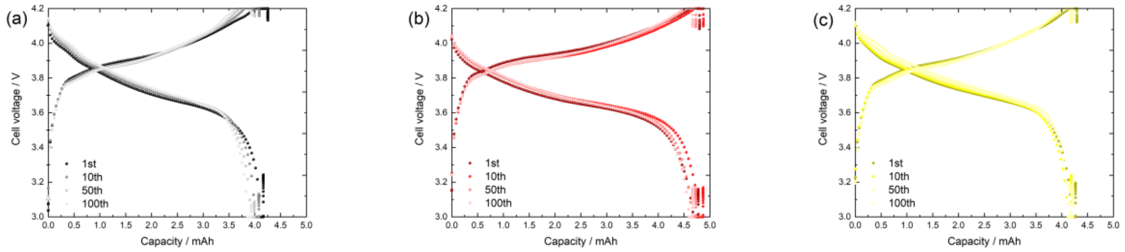

Figure S12: Typical charge/discharge curves (cell voltage vs. capacity) for the LIBs with graphite/LCO AMs with (a) foil CCs, (b) Cu-MWGG-1 / Al-MWGG CCs, and (c) Cu-MWGG-2 / Al-MWGG CCs. Shown are the data from the 1st, 10th, 50th and 100th cycle.

Figure S12 shows typical charge-discharge curves (cell voltage vs. capacity) for the three cell-types with LCO as AM (*cf*. Figure 7g).

Reference

[1] R. Schmuch, R.Wagner, G. Hörpel, T. Placke, M. Winter, *Nat. Energy* **2018**, 3, 267.

[2] M. Kane, Tesla's 4680-Type Battery Cell Teardown: Specs Revealed, https://insideevs.com/news/598656/tesla-4680-battery-cell-specs/ **2022**, accessed 03 May 2023.

[3] E. Hornbogen, *Int. Mater. Rev.* **1989**, 34, 277.

[4] M. Avisar-Levy, O. Levy, O. Ascarelli, I. Popov, A. Bino, *J. Alloys Compd.* **2015**, *635*, 48.
